# GRADE IT—A Literacy-Based Assessment Tool for *Generating Research-Based Assessment Data to Evidence the ImpacT of Anti-doping Education* via Athletes' Capability to Make the Right Decision

**DOI:** 10.3389/fspor.2022.842192

**Published:** 2022-03-15

**Authors:** Cornelia Blank, Katharina Gatterer, Marie Overbye, Wolfgang Schobersberger, Bernhard Streicher, Andrea Petróczi

**Affiliations:** ^1^Institute for Sports Medicine, Alpine Medicine and Health Tourism, UMIT - Private University for Health Sciences, Medical Informatics and Technology, Hall in Tirol, Austria; ^2^Faculty of Health Sciences and Sport, University of Stirling, Stirling, United Kingdom; ^3^Tirol Kliniken GmbH, Innsbruck, Austria; ^4^Department of Psychology, Ludwig-Maximilians-University, Munich, Germany; ^5^School of Life Sciences, Pharmacy and Chemistry, Kingston University, Kingston upon Thames, United Kingdom

**Keywords:** anti-doping education, literacy, evaluation, decision-making, junior elite athletes

## Abstract

The International Standard for Education (ISE) mandates Code Signatories to plan, deliver, and evaluate anti-doping education. Performance-based evaluation of anti-doping education requires alignment between educational goals, content, and defined outcomes. Based on an existentialist teaching and learning philosophy, we aimed to develop and test an anti-doping impact evaluation tool, to assess the impact of anti-doping education on doping awareness, literacy (DAL), perceived trust, and legitimacy. We propose that the impact of anti-doping education is best captured through assessment of situation-specific (social) cognitive mediators of actions that influence athletes' choices in the context of sport-related goals. In phase one, we aimed to develop and test the Generating Research-based Assessment Data to Evidence the ImpacT of anti-doping education (GRADE IT) evaluation tool that comprised a set of social cognitive components: anti-doping knowledge, DAL, perceived trust, and legitimacy of anti-doping (organizations). In phase two we assessed whether anti-doping education impacts knowledge, the three DAL stages (functional, interactive, and critical literacy), perceived trust and legitimacy. Phase one enrolled 986 junior elite athletes, and we showed that all GRADE IT components performed well. After revision of the tool for phase two, we validated the assumption that anti-doping education impacts the likelihood that athletes will make the “right” choice (based on a new set of data from 1,255 junior elite athletes). Comprehensive education was associated with higher scores for all stages of DAL, as well as perceived trust and legitimacy. Even athletes reporting no education had positive scores for all included outcomes, supporting the assumption that most athletes wish to engage in clean sport behaviors and might need anti-doping education not to prevent them from doping, but rather to reinforce their commitment to clean sport. In conclusion, GRADE IT, which is available in 23 languages, is a suitable tool for application to young, emerging athletes to satisfy the ISE requirement for evaluating anti-doping education programs. Researchers and practitioners alike are advised to collect additional data to further validate the tool for adult athletes, and to apply it longitudinally to identify if changes in doping prevention policies have a delayed effect on DAL, perceived trust, and legitimacy.

## Introduction

Using education to raise awareness, inform and communicate is one of the main aims of the World Anti-Doping Agency's (WADA) strategy to prevent intentional and unintentional anti-doping rule violations (ADRV; World Anti-Doping Agency, 2021c). Prevention, as defined in a public health context (Hurrelmann et al., 2009), focuses on identifying risk factors for a certain outcome, with the aim of minimizing these factors and decreasing the likelihood of undesired outcomes. Accordingly, most behavioral anti-doping research conducted in the last two decades focused on identifying risk factors for doping behavior (Ntoumanis et al., 2014; Blank et al., 2016), with the long-term aim of establishing an evidence base as a foundation for preventive measures to eliminate risk factors. Despite this growing body of research, resulting evidence-based preventive strategies have apparently not been implemented (Gatterer et al., 2020). Gatterer et al. (2020) review showed that a significant proportion of anti-doping organizations limits their anti-doping education to information provision, with only a few National Anti-Doping Organizations (NADOs) offering comprehensive programs considering both risk and protective factors. This limited focus, however, may not hinder the impact of the programs, because a large proportion of athletes would not dope anyway due to their values and upbringing (Petróczi et al., 2021a; Shelley et al., 2021). The athletes in these studies had already decided to be compliant with anti-doping rules before receiving any anti-doping education, yet the dominant approach to anti-doping education remains preventive, and assumes that athletes need to be “saved” from doping. As such, evaluating the success of anti-doping education solely based on a decrease in the incidence of ADRV—which is largely determined by detection and sanctioning—is neither sufficient nor appropriate.

Additionally, there is evidence of a lack of alignment between learning outcomes (which are evidence-based) and the elements of existing anti-doping education (Woolf, 2020), which might explain the seemingly unsuccessful education initiatives. Moreover, these learning outcomes are not aligned to WADA's definition of doping. In detail, most research assessing risk factors for doping behavior define it as the use of prohibited substances and/or prohibited methods (Ntoumanis et al., 2014; Blank et al., 2016), which only refers to two of the 11 ADRV defined by the World Anti-Doping Code (WADC; World Anti-Doping Agency, 2021c). Some exceptions include Chan et al. (2019) and Chan et al. (2020).

From the perspective of content, research suggests that variables identified as risk factors (Ntoumanis et al., 2014; Blank et al., 2016) are in fact a mixture of risk factors for doping (Petróczi et al., 2017; Gatterer et al., 2019) and protective factors against doping (Overbye et al., 2013; Erickson et al., 2015; Englar-Carlson et al., 2016). This is problematic, as it has been argued that reasons to dope (risk factors) and reasons not to dope (protective factors) pertain to two distinct goals and thus cannot be considered as the opposite of each other (Overbye et al., 2013; Petróczi et al., 2017, 2021a; Gatterer et al., 2019). This problem was also highlighted by a recent synthesis of qualitative research on barriers to, and factors promoting, clean sport (Williams et al., 2021). The authors of the review outlined the importance of knowledge as both an “enabler” and barrier to doping, and highlighted the lack of understanding of the complexity of motivation, which involves both physical and psychological capabilities. It was further argued that motives to dope were mostly associated with functional reasons, such as the pressure to win, fear of losing sponsors, and preventing a loss in performance when injured (Whitaker et al., 2017; Gatterer et al., 2019).

Further research highlighted that risk factors must be considered on multiple levels (including structural risks) in the interactions between the environment and individual (Petróczi and Aidman, 2008; Petróczi, 2013; Petróczi et al., 2017). For example, it has been suggested that sports environments are “dopogenic,” with multiple influencing factors at the local level and structural factors interacting to increase risks for athletes (Backhouse et al., 2018). Moreover, elite sport has been characterized as a risky occupation with unique features influencing interactions and interdependencies between individuals and their environment (Overbye, 2018). While these approaches focus on prevention, they also support the need for empowering athletes to cope with the pressures and risks posed by different environments.

Based on the above research, and as already discussed by Petróczi et al. as part of the Erasmus+ projects Safe You+ (Petróczi, 2018) and RESPECT (Petróczi et al., 2021a), it may be time to rethink the theoretical foundations of current anti-doping education approaches, by transitioning from an epidemiological and psychological perspective to one of health promotion and literacy. In health sciences, health promotion and literacy perspectives play an important role in the ability to stay healthy while facing health risks. Translated to doping, this would mean the ability to remain committed to clean sport behavior and compliant with anti-doping rules, which are two dissociable dimensions (Clancy et al., submitted) despite both being associated with the pressure of the sporting system. Judicious decision-making is a significant concept in health literacy arising from the paradigm of health promotion. If intentional doping is often a coping strategy (Petróczi and Aidman, 2008), as much of the evidence tends to support, it can be considered a decision, i.e., a deliberate choice. It may be a “bad” decision from anti-doping and health perspectives, but is nonetheless something that athletes decide to do, irrespective of whether it is based on a thorough risk-benefit analysis. Anti-doping education should thus address this decision-making process, to help athletes make the “right” decision according to their specific context and circumstances by increasing their literacy.

### Concepts of Health Promotion and Health Literacy

Health promotion is “the process of enabling people to increase control over, and to improve their health,” as defined by the World Health Organization's Ottawa Charter (World-Health-Organization, 1986. p. 1). Health promotion activities aim to increase resources, capacities, and abilities, to empower individuals to make the right choices in terms of health. Making the right choice is in turn closely related to the concept of health literacy. Nutbeam (2000, p. 263), defines health literacy as an outcome of health education, and more specifically as the “personal, cognitive, and social skills which determine the ability of individuals to gain access to, understand, and use information to promote and maintain good health.” An essential role in achieving the desired conditions and applying newly acquired knowledge is played by self-efficacy. In detail, Nutbeam (2000) specifies three types of literacy, which are assumed to build upon each other and ultimately lead to greater empowerment: (a) basic/functional literacy (i.e., factual information), (b) communicative/interactive literacy (i.e., developing personal skills based on knowledge), and (c) critical literacy (i.e., information on social and economic determinants of health important to achieve policy changes). These three types of literacy have also been shown to be associated with several health-related outcomes, including compliance with prescribed therapeutic regimes (Ad Hoc Committee on Health Literacy for the American Councol on Scientific Affairs, 1999; Nutbeam, 2000).

### Applying the Health Literacy Concept to Anti-Doping

**Figure 1:**
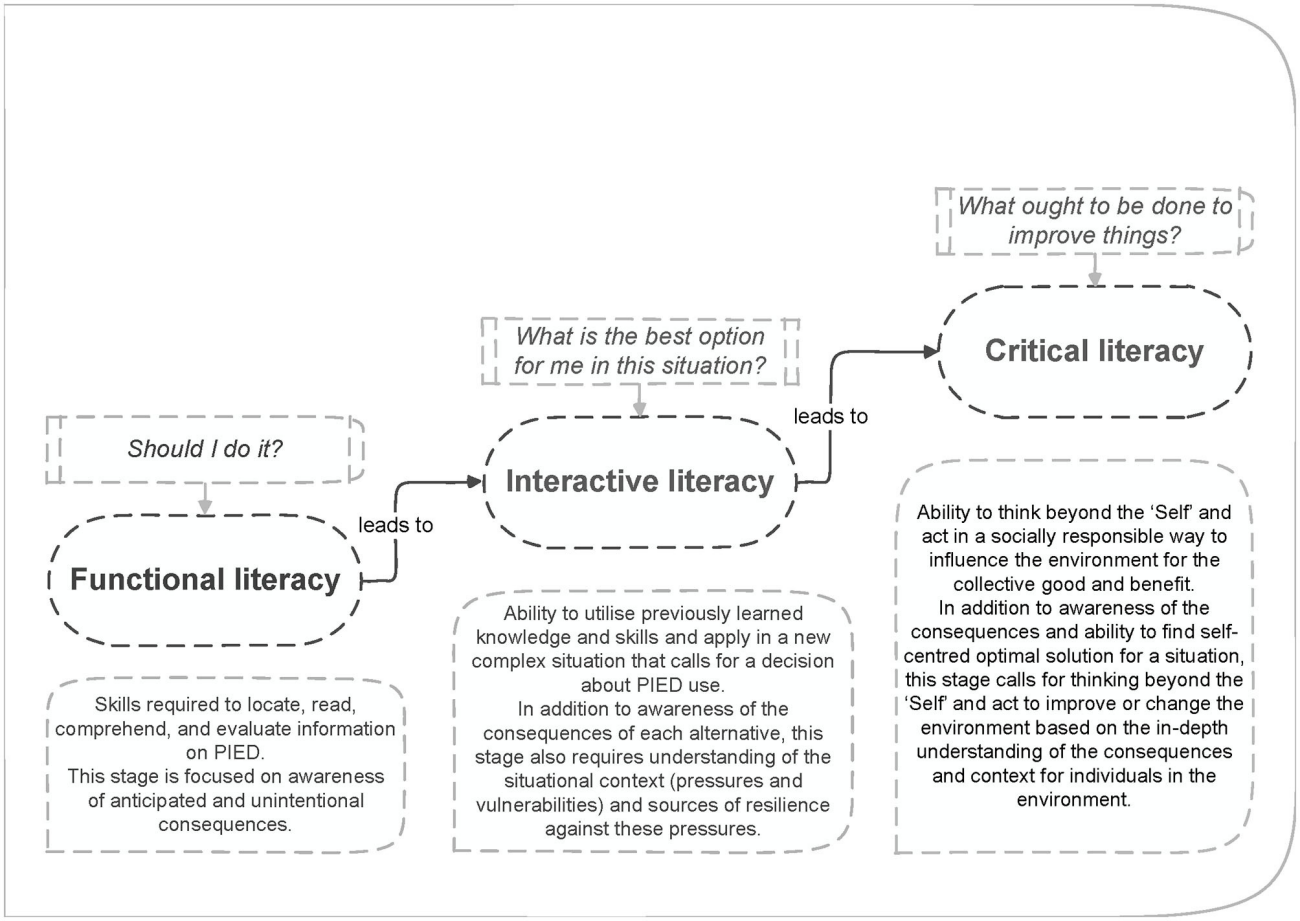
Schematic diagram of the Doping Awareness Literacy (DAL) stages.

We argue that applying the health literacy concept to the doping context is a promising approach to enable elite athletes (competing at international level) to make the right decisions. The concept of Doping Awareness Literacy (DAL; see Figure 1), was originally developed by Andrea Petróczi and her team at Kingston University for the EU ERASMUS+ funded “SAFE YOU” project (safeyou.eu), and directly built on the health literacy concept applied to performance-enhancing drugs in competitive sport. This initial model captures the process of DAL development in three distinctive stages: Knowing (Functional Literacy), Deciding (Interactive Literacy), Leading (Critical Literacy). The DAL model is progressive and incremental, and aims to strengthen self-efficacy to promote informed decisions about the use of performance- and image-enhancing drugs (PIEDs) for better self-care and health (direct and indirect outcomes, respectively; Petróczi et al., 2021b).

Vamos and Steinmann (2019) also discussed the idea of health literacy in the context of youth sport in Germany; however, they did not directly link it to doping. Their focus was on improving health literacy in young professional sportspeople, to decrease the potential harm caused by pursuing professional sport (where doping is only one of several possible health risks). Vamos and Steinmann (2019) argued that health literacy can be considered an asset that might help sportspeople overcome personal and structural barriers to health within the context of elite sport, and enhance control over social, environmental, and economical health determinants.

The International Standard for Education (ISE) mandates Code Signatories to plan, deliver, and evaluate anti-doping education initiatives (World Anti-Doping Agency, 2021b); this does not directly address doping literacy, but it is considered key that education programs help athletes make ethical decisions. According to the Guide to the ISE (World Anti-Doping Agency, 2021a), making an informed decision involves values, awareness, information, and anti-doping education (in terms of Code compliance). From a doping literacy perspective, information and awareness are important as a basis for increasing Functional Literacy, whereas anti-doping education is necessary for Interactive Literacy, which reflects the development of skills and capabilities to handle specific situations. Lastly, even though not explicitly stated, the values component of the ISE can be considered as part of Critical Literacy, because it extends beyond the athlete him/herself and to encompass the environment, culture and values.

The ISE mandates that organizations with responsibility for anti-doping develop a plan for evaluating and demonstrating the effectiveness of their anti-doping education. This point was cited as a barrier to the implementation of anti-doping education by Gatterer et al. (2020). In detail, a representative from Asia stated that “Lacking proper method of education evaluation would be a setback to justify the effectiveness of doping prevention programs, which could hinder it from getting more attention and funding from the stakeholders,” and a representative from Europe cited the lack of ability to demonstrate impact (Gatterer et al., 2020. p. 235). Thus, organizations responsible for anti-doping education must present a plan pertaining to the anti-doping education measures that they are aiming to implement, and how they intend to assess their effectiveness. A tool to assess the effectiveness of anti-doping education, in terms of a decrease in ADRV, is lacking. Generally, evaluating prevention initiatives is methodologically challenging, especially if the outcome cannot easily be measured—as is the case with ADRVs. However, assuming similar associations between DAL and clean sport behavior, as well as commitment to Anti-Doping rules, it would be worthwhile to develop a tool for measuring associations between anti-doping education and the stages of DAL. Such a tool could be used for longitudinal studies to observe changes and connect them to possible changes in doping prevention strategies (e.g., the introduction of new rules and national legislative approaches, etc.).

### Aims

The current study is a part of a larger project funded by the International Olympic Committee (IOC). The project's overall aim is to explore current anti-doping education and its possible effects. Whereas the previous phase of the study focused on the provider (Gatterer et al., 2020) and consumer sides (Gatterer et al., 2021) of anti-doping education, the primary goal of the current study was to develop and test a tool for assessing the expected outcomes of the anti-doping education provided by NADOs and International Federations (IFs) under the ISE. We operationalized these outcomes through the DAL stages of awareness, knowledge, and self-efficacy. We also expected anti-doping education, even though it is not a direct education goal, to influence the perceived legitimacy of anti-doping and perceived trust of the involved organizations. As described in detail below, we approached these aims in a stepwise fashion.

### Conceptual Framework for Developing the Evaluation Tool

There are multiple ways to evaluate anti-doping education, each presenting its own challenges (Petróczi et al., 2017). This project is based on existentialist teaching and learning philosophy. Stemming from Lawless' succinct statement that there are “no universal standards for a human life: we are what we do, the sum of our actions” (Lawless, 2005, p. 326), existentialist teaching and learning philosophy promotes learner agency, and aims to empower learners to make their own decisions, as opposed to dictating to them as to what they should and should not do. In this framework, anti-doping rules are a set of conditions that regulate participation in competitive sport, just like the rules of a specific sport. In line with this approach, we view the role of anti-doping education as enhancing athletes' skills and capacity for knowledge and understanding, and to then apply these rules for authentic choices and behavioral conduct. Consequently, effectiveness is not primarily evaluated according to the number of athletes resisting doping, although better awareness and knowledge of the rules is expected to reduce the number of unintentional ADRV. Instead, we propose that the impact of anti-doping education is best captured through situation-specific (social) cognitive mediators of actions (Bandura, 1986, 2001) influencing athletes' choices in the context of sport-related goals, expected outcomes and socio-structural constraints (such as barriers and opportunities associated with particular sport settings, as well as political, economic, or environmental systems).

**Figure 2:**
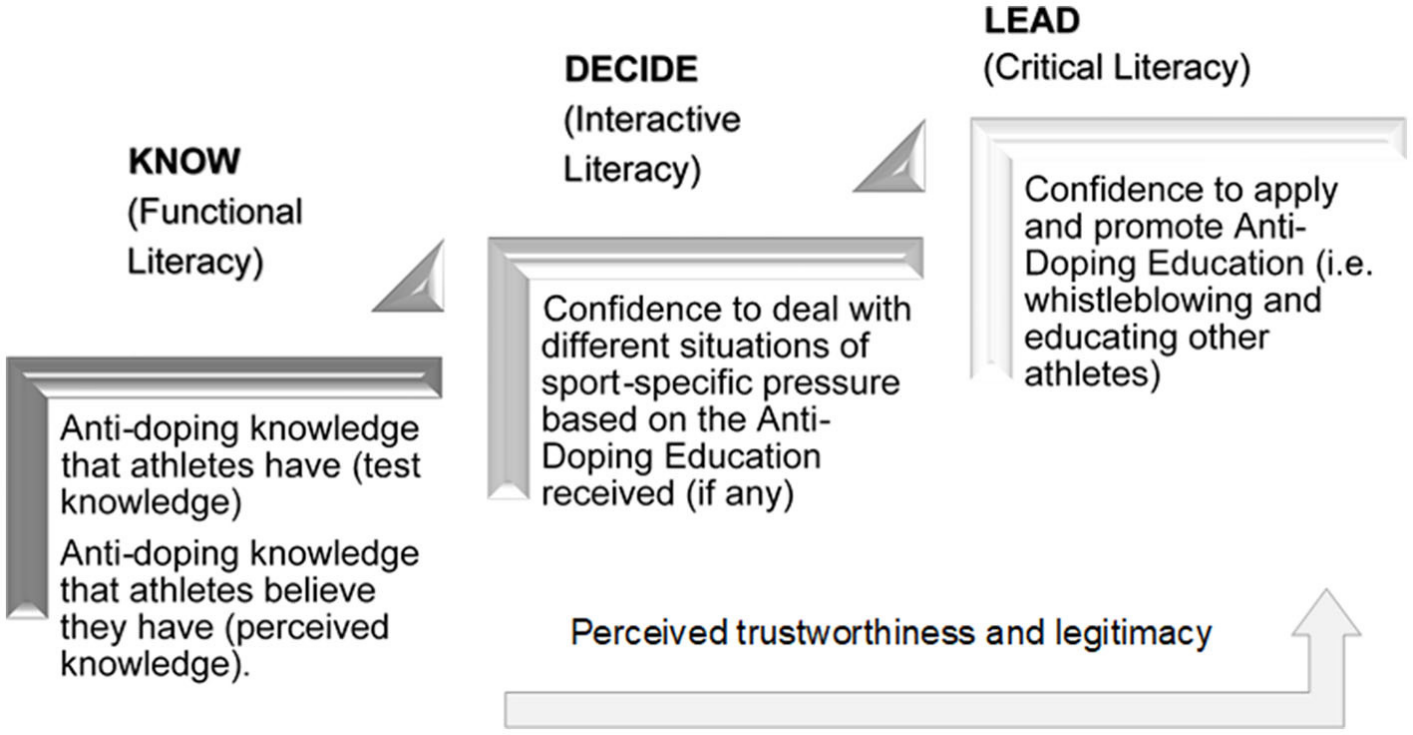
Stages of DAL and associated skills.

Based on a substantial body of evidence from health science on the associations among health literacy, perceived trust and the legitimacy concepts outlined above, we infer that these concepts are also a proxy for making the right decisions in the context of anti-doping. As such, we are especially interested in the question of whether anti-doping education influences athletes' DAL, as well as trust and legitimacy perceptions (Woolway et al., 2020). We hypothesized that anti-doping behavior can be promoted by increasing awareness and literacy. Using the DAL model, Figure 2 outlines the skills and knowledge we expect to be accrued in each stage of effective anti-doping education.

The aim of anti-doping education is to enable athletes to make informed decisions related to clean sport principles and performance enhancement, and to be able to adhere to anti-doping rules. To make such decisions, athletes need awareness of the problem, and the capability and ability, i.e., the literacy, to make the right decision to address it. It is important that this process starts early, i.e., at the beginning of an athlete's career, as they appear to be more prone to risky behavior and peer pressure during adolescence, when critical thinking develops (Flammer and Alsaker, 2002; Oerter and Dreher, 2008; Steinberg, 2016). This change in perspective does not necessarily equate to a change in current doping prevention initiatives. Even before the implementation of the ISE, information- and values-based education were key pillars of doping prevention, even though a recent study analyzing 53 National Anti-Doping Organizations with respect to their prevention measures revealed that the majority were mostly concerned with information (Gatterer et al., 2020).

### The Role of Legitimacy, Trust, and Trustworthiness

Perceived legitimacy of authorities, such that their actions are regarded as proper, just, and appropriate (Tyler, 2006), is considered an important factor in anti-doping rule compliance. Related concepts such as trustworthiness (an attribute of the “object,” e.g., WADA as organization can be trusted) and trust (an attribute of the “person,” e.g., I, as an athlete, trust WADA) also play an important role in the decision-making process about compliance, and how athletes feel about being compliant (Woolway et al., 2020). Although legitimacy is not the same as trustworthiness, nor perceived legitimacy equates to trust, but they are closely related concepts. Especially in the context of doping, trust in anti-doping organizations cannot be operationally defined without the ingredients of legitimacy. Legitimacy is what organizations are set out to do (that is what should work in principle); trustworthiness is a characteristic of this organization judged on past and present conducts, and trust (by a person) is an anticipation of what organizations will actually do in a specific context. Dreiskämper et al. (2016) trustworthiness scale for anti-doping organizations is based on the three pillars of trust—ability, benevolence, and integrity—that were originally proposed by Mayer et al. (1995)—and showed its importance in anti-doping.

In the context of health science, the positive association between health literacy and compliance was shown to be affected by the level of trust in the health provider (Mancuso, 2010; Naghavi et al., 2019), as well as in the medical establishment (Bender and Bender, 2005). These concepts are expected to be similarly relevant to doping science. In support of this, Shelley et al. (2021) showed that clean athletes could be strong advocates for doping prevention, but only if they trust the current system. In support, Woolway et al. (2020) suggested that a low level of perceived legitimacy of anti-doping rules and organizations might result in a low level of compliance with the anti-doping system and called for research about the role of anti-doping education in perceived legitimacy. Additionally, Petróczi et al. (2021a) concluded that the inequalities and unfairness of the doping control system (an aspect of perceived legitimacy), as perceived by elite athletes, undermine trust in anti-doping and that this issue should be acknowledged and addressed.

### Stepwise Approach of the Aims

#### Phase One

The aim of phase one was to develop an effective assessment tool with high content validity, in collaboration with internationally renowned experts in anti-doping with different research backgrounds. We named this tool GRADE IT, standing for “Generating Research-based Assessment Data to Evidence the ImpacT of anti-doping education;” and included markers/variables that we identified as relevant for promoting clean sport behavior or preventing anti-doping rule violations (linked to the outcomes of current doping prevention initiatives, as identified by desktop research; Gatterer et al., 2020); these variables map onto the DAL stages. The markers are intended to be sufficiently sensitive to detect changes in doping prevention strategies, as well as evidence-based. The research questions for phase one were as follows: (a) how appropriate is the item difficulty (for the knowledge test questions) and is the discriminatory power sufficient? (b) are the markers sensitive to changes? and (c) is anything missing that should be added based on (a) the feedback of the athletes questioned and (b) new developments in doping prevention initiatives? The research project started in 2017, when the development of the ISE was in progress but not yet implemented. Thus, a secondary goal was to refine GRADE IT not only based on the results of the first study, but also on the progress of the ISE.

#### Phase Two

The aim of phase two was to assess whether anti-doping education makes a difference to the three literacy concepts of Functional, Interactive and Critical Literacy, as well as to the concepts of perceived trust and legitimacy, by surveying athletes using the assessment tool refined on the basis of phase one.

## Materials and Methods

### Participants

Participants were recruited at the Youth Olympic Games (YOG) in Buenos Aires (2018) and European Youth Olympic Festival (EYOF) in Sarajevo (2019) (for phase one), and at the EYOF Baku (2019) and YOG Lausanne (2020) (for phase two). The athletes completed the questionnaire at the sport events, on tablets or computers, or on their own mobile devices (using a QR code). Participation was anonymous and voluntary. Informed consent was provided by all athletes prior to completing the questionnaire. For more details on participant recruitment, please refer to Gatterer et al. (2021). The study was approved by the ethics board of the first author's university (RCSEQ 2444/18).

### Procedure and Data Collection

**Figure 3:**
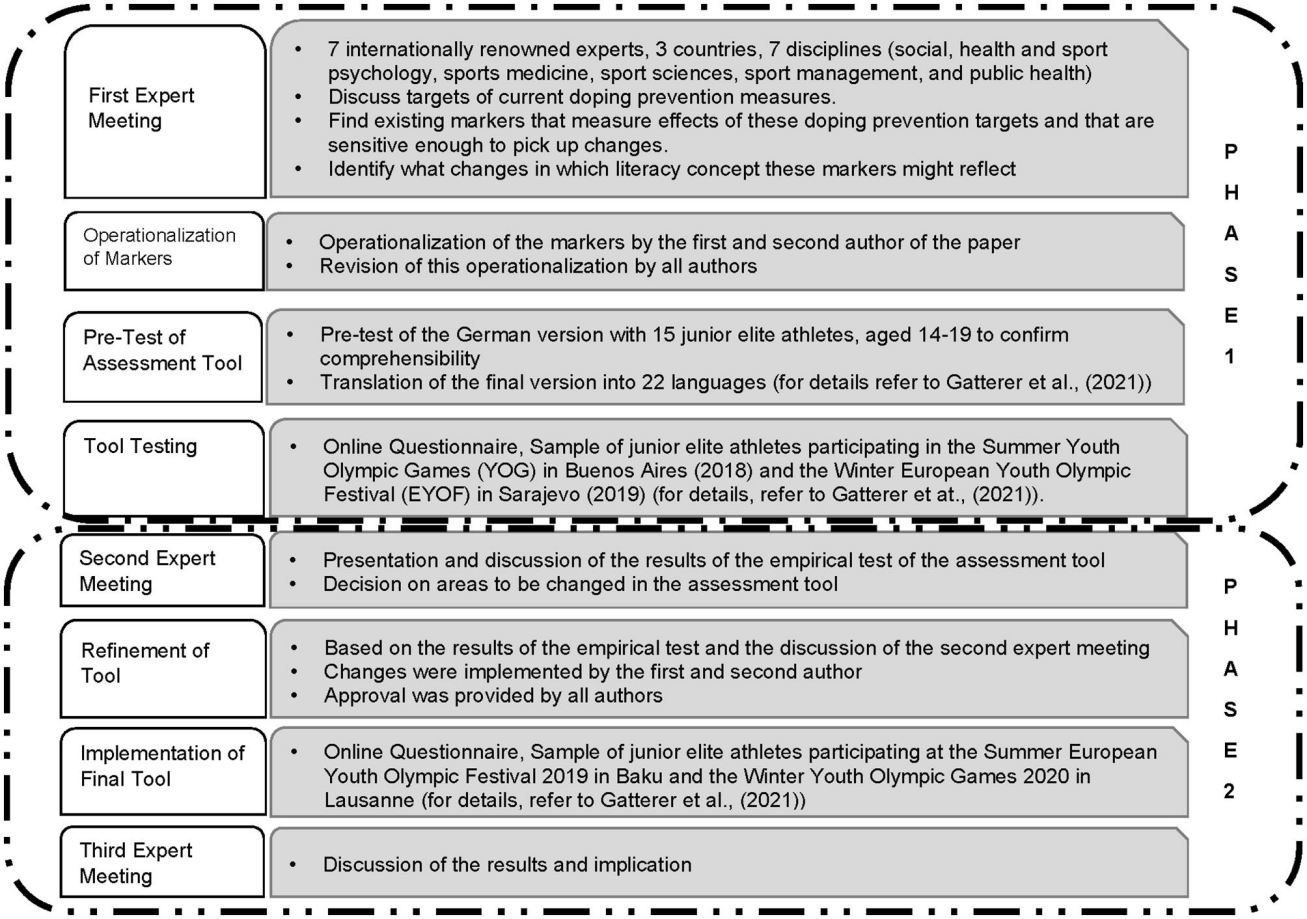
Description of the procedure and data collection for phases one and two.

The procedure of the two phases is detailed in Figure 3. Details are also provided as part of the results for phase one, as this phase focused on the development of the tool. The two versions of the tools are attached as Supplemental Materials 1, 2; differences between the two versions are highlighted in yellow.

Empirical data were collected by distributing the tool online to the junior elite athletes (accessible *via* tablets and QR codes for their phones). To connect with the athletes, several “gatekeepers” were used [Chef de Mission Seminar, Medical Meeting, Young Ambassadors, and direct contact with the Youth Olympic Villages; for details, refer to Gatterer et al. (2021)]. Athletes also made verbal comments (at random) through the tool, which we recorded as notes. This feedback was not thematically analyzed but considered in the discussions at the expert meetings.

### Data Analysis

Data from GRADE IT were analyzed using SPSS software [(version 26); IBM Corp., Armonk, NY, USA]. Data were analyzed descriptively and are presented as frequencies, means and standard deviation. To assess item difficulty (for the knowledge test using a true-false format) and the discriminatory power of the answer options (for Likert scale questions), frequencies (test questions), and mean and standard deviations (Likert scale responses) were computed. To determine whether the markers are sensitive enough to detect changes in doping prevention strategies focusing on anti-doping education, and depending on the heterogeneity of variance, a univariate analysis of variance (ANOVA) or Welch test was used to compare outcome variables among groups of athletes receiving different levels of education. *Post-hoc* tests were applied to significant results (Bonferroni or Tamhane) to determine the groups that were different. The level of significance was set at *p* < 0.05.

## Results Of Phase One—Tool Development And Testing

### Step 1: First Expert Meeting

In total, seven internationally renowned experts, mostly senior researchers in their respective fields with 2–20 years (or more) of experience in anti-doping research (for details, refer to Figure 3), formed the expert panel. Five of the seven experts participated in the meeting held in Hall in Tyrol in 2017, and two experts, who could not personally attend the meeting, discussed it thereafter. Those five experts (present in Hall in Tyrol in 2017) continued to be involved throughout 2019, and four of these experts (co-authors of this paper) participated in the last expert meeting in 2020 (for details, refer to Figure 3). Regarding motives to dope or not dope, the expert panel agreed (based on scientific evidence) that athletes are likely to dope for functional reasons (i.e., coping with specific pressures), whereas motives not to dope are value-based (social, cultural, attitude, and norms; Gatterer et al., 2019). As it cannot be assumed that most athletes dope (especially at the age of 14–19 years), both motives to dope and refrain therefrom should be considered, even though they are not explicitly distinguished within GRADE IT. Given the lack of alignment of the content of anti-doping education with the intended learning outcomes (Woolf, 2020), as supported by Gatterer et al. (2020), and the expert group opinion that the implementation of value-based education is lacking, it seemed unlikely that variables or constructs reflecting values (i.e., attitudes, norms etc.) would be sensitive to changes in DAL, perceived trust or legitimacy, simply because they are not included in current doping education programs. Therefore, we decided to focus on the components of the ISE aligned to the stages of DAL (refer to Figure 2) including knowledge, confidence to deal with pressure and confidence to apply and promote anti-doping education (i.e., whistleblowing and education of other athletes), as these reflect the levels of the DAL, as well as perceived legitimacy and trust (further outlined in Table 1). Based on its content, GRADE IT can be considered as an anti-doping education impact evaluation tool.

**Table 1:** Markers to be included in GRADE IT.

**Level of DAL**	**Marker/variable: content**	**Measures' status and changes of effects of:**
**Markers that directly reflect anti-doping literacy**		
Functional DAL	Functional DAL includes information on the anti-doping knowledge of athletes, including self-assessed (perceived) knowledge and knowledge based on test questions.	Information-based prevention
Interactive DAL	Interactive Literacy includes questions on how to deal with pressure. Athletes who indicated that they had received education on dealing with pressure were asked about the education received. Those who did not receive any education were asked about their confidence in dealing with sport-specific pressures.	Education prevention strategies based on informed decision-making
Critical DAL	Critical Literacy includes questions pertaining to whether athletes feel confident in applying and promoting doping prevention measures. In detail, athletes were questioned about whistleblowing and the education of other athletes.	Education prevention strategies and anti-doping policies
**Markers that might affect compliance with DAL**		
Trust in organizations	Might inform Interactive and Critical DAL. Athletes might be more compliant if they consider anti-doping organizations trustworthy. This goes beyond capability, and might influence the actual decisions of an athlete/their willingness to show compliant behavior.	Anti-doping policy
Legitimacy	Might inform Interactive and Critical DAL. Athletes might be more compliant, and feel happier about this, when there is a high level of perceived legitimacy with respect to the rules. This goes beyond capability, and might influence the actual decision of an athlete to show compliant behavior.	Anti-doping policy

### Step 2—Operationalization of the Markers

The operationalization of the variables was further discussed, and a decision was made that the wording should be simple, and that questions must be specific (i.e., no open-ended questions). In addition to the markers, GRADE IT also captures socio-demographic information and assesses whether athletes have received anti-doping education. Respondents can indicate the content of any education received; for data analysis purposes, three mutually exclusive groups were distinguished based on this variable: “no education,” “information,” and “comprehensive education”—the latter two groups are based on the classification of Gatterer et al. (2020).

#### Measures

##### Socio-demographic Characteristics

The online questionnaire captured socio-demographic information (age, gender, sport, and country), and whether the athletes had experience with anti-doping education. Athletes with anti-doping education had to indicate the content thereof, which was then classified into three groups: no education, information, and comprehensive education [for details, refer to Gatterer et al. (2020)].

##### DAL—Functional Literacy

Nutbeam (2000), defined functional literacy as “factual knowledge” and based on the DAL concept that translated the concept of health literacy to the doping context (Petróczi et al., 2021b), the stage of Functional Literacy was described with “knowing.” Therefore, we operationalized Functional Literacy as objective test knowledge of rules and responsibilities under the WADC as well as the level of perceived factual knowledge for the purpose of this paper. Test knowledge was measured using 10 items pertaining to roles and responsibilities (according to the WADC 2015) and three items on the consequences of a positive doping test. Responses were recorded using a “True”/“False”/“Cannot assess” format. Perceived knowledge was indexed by nine items pertaining to how well-informed athletes are about the rules and responsibilities specified in the WADC 2015 (nine items in total). A Likert response scale was used, with the additional option of “Cannot assess” (for the English version of the initial questionnaire, refer to Supplementary Material 1).

##### DAL—Interactive Literacy

The self-efficacy section, designed to represent Interactive Literacy, comprised five items pertaining to various forms of pressure [e.g., “physical limitations (injuries, illness, fatigue, and overtraining”)] and how confident they felt in dealing with them without using prohibited substances and/or methods. Athletes who indicated that they had received education were then asked how confident they felt based on that education. The response scale was a Likert scale with the additional option of “Cannot assess” (for the English version of the initial questionnaire, refer to Supplementary Material 1).

##### DAL—Critical Literacy

Based on the DAL concept that translated the concept of health literacy to the doping context (Petróczi et al., 2021b), the stage of Critical Literacy was described with “leading” and includes the “ability to think beyond the self” to “influence the environment.” Therefore, we operationalized Critical Literacy as the perceived confidence to act and influence the direct environment in a positive way with respect to anti-doping. Critical Literacy was indexed by two items with a “True”/“False”-format pertaining to whether athletes felt confident about educating other athletes and reporting doped athletes, based on the education that they had received (for the English version of the initial questionnaire, refer to Supplementary Material 1).

##### Perceptions of Anti-doping Legitimacy and Trust

Anti-doping legitimacy was assessed by three statements covering normative (anti-doping is important because it protects clean sport, which is worth protecting) and procedural (fair process and fair outcome) legitimacy (Woolway et al., 2020). The perceived trust of different organizations was assessed using three statements pertaining to ability, benevolence and integrity, as important factors in perceived trust (Mayer et al., 1995) also applied by Dreiskämper et al. (2016) in the context of anti-doping. In this study, ability was operationalized as trust in the capability of an organization to fulfill its designated role; benevolence referred to the concern of the organization for its members and integrity (keeping of promises). A Likert response scale with the additional option of “Cannot assess” was used again (for the English version of the initial questionnaire, refer to Supplementary Material 1).

Since the athletes' ranged in age from 14 to 19 years, the final wording of the questions was discussed with an expert in developmental psychology, to ensure age-appropriateness. The initial GRADE IT is included in the Supplementary Material 1.

#### Steps 3 and 4: GRADE IT (Pre-) Testing

##### Description of Sample and Level of Education Received

Prior to implementation, the survey was pilot-tested on 25 Austrian junior professional athletes aged 14–19 years, which did not result in any additional changes to GRADE IT. Following the pilot testing, in phase one, 968 athletes fully completed the survey (19.8% of all athletes attending the events) and were thus included in the data analyses. The socio-demographic characteristics are shown in detail in Table 2.

**Table 2:** Socio-demographic characteristics of the study cohort—phase one.

	***n***	**%**	
**Event**
Buenos Aires (Summer YOG)	468	48.3	
Sarajevo (Winter EYOF)	500	51.7	
**Origin**
Europe	648	68.1	
Asia	80	8.4	
North America	30	3.1	
South America	101	10.4	
Africa	63	6.5	
Oceania	30	3.1	
**Gender**
Female	477	49.3	
Male	435	44.9	
**Sport**
Individual	670	69.2	
Team	215	22.2	
**Member of RTP**	253	26.1	
**Gender**
Male	435	44.9	
Female	477	49.3	
	**Mean**	**SD**	**Min-Max**
**Age (y)**	16.62	0.93	14–18

*% values do not all sum to 100% due to missing data*.

*YOG, Youth Olympic Games; EYOV, European Youth Olympic Festival; RTP, Registered Testing Pool*.

**Figure 4:**
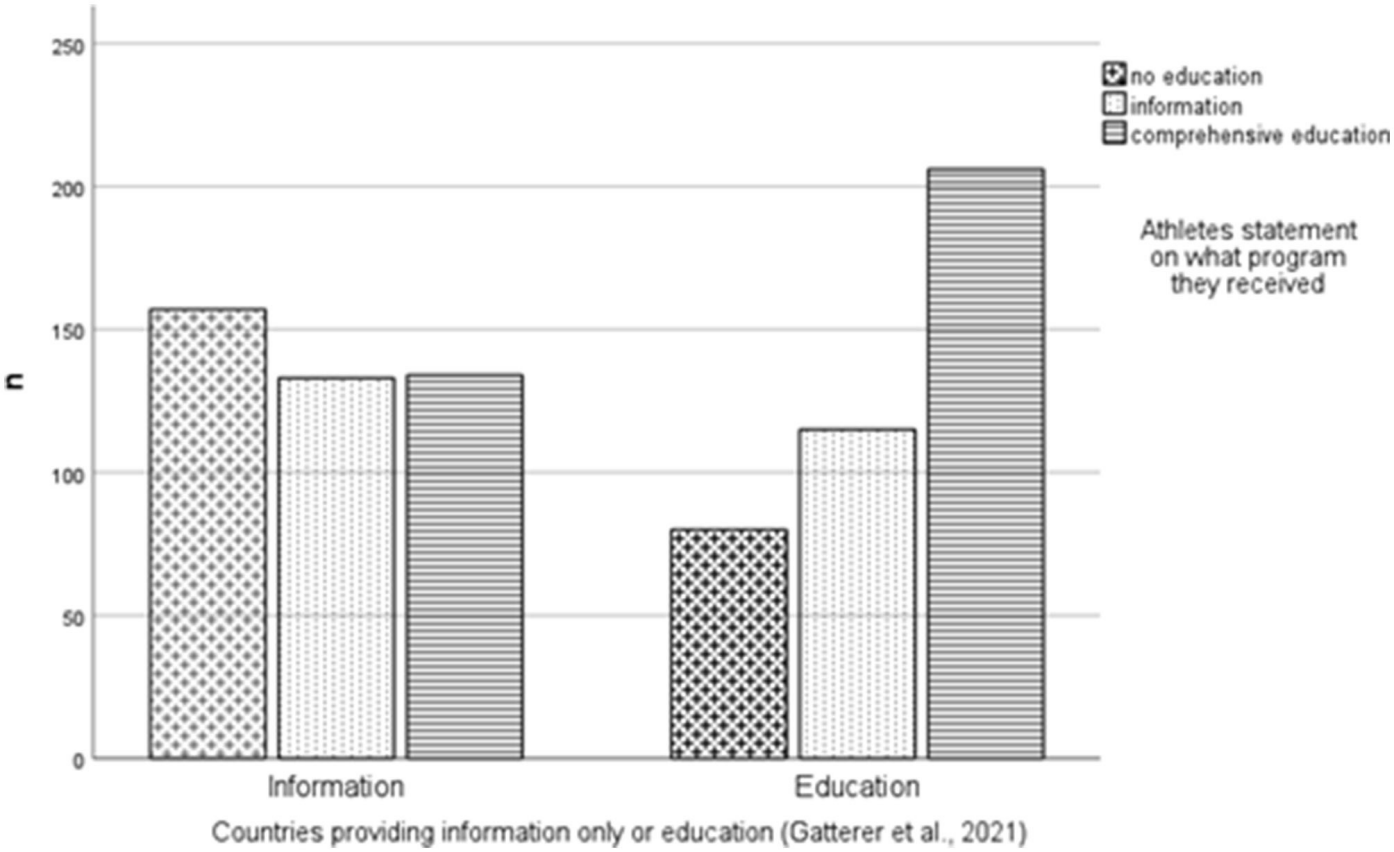
Distribution of perceived education of athletes from countries providing either information or education. Chi square: 41.37, *p* < 0.001, Phi: 0.224.

Of the 968 elite youth athletes, 28.9% had never received anti-doping education, 32.4% received information, and 38.4% received comprehensive education; 0.2% did not provide an answer. Based on the country classification of Gatterer et al. (2021), 44% of the athletes (*n* = 426) originated from countries whose NADOs provided information only, and 41.4% (*n* = 401) were from countries whose NADOs provided comprehensive education. For 14.6% (*n* = 141) of the athletes, the country data were missing. Of the 426 athletes from countries whose NADOs technically provide information only [as defined by Gatterer et al. (2020)], 37.3% (*n* = 158) indicated that they had never received any information, while 31.6% (*n* = 134) indicated that they had received more than information only, i.e., comprehensive education (e.g., role plays). Of the 401 elite youth athletes from countries whose NADOs technically provide comprehensive education [as defined by Gatterer et al. (2021)], 20% (*n* = 80) indicated that they had never received any education and 27.7% (*n* = 115) indicated that they had only received information. Chi square analysis revealed significant incongruence, i.e., participants from countries providing comprehensive education also reported receiving information only, and no education. Also, some participants from countries providing information only reported receiving no education, but also comprehensive education (for details, refer to Figure 4).

##### Discriminatory Power of Items and Item Difficulty

Descriptive statistics for each individual item for Functional and Interactive Literacies, as well as for perceived trust and legitimacy, are displayed in Supplementary Tables 3, 4. To allow for comparison, the scores for test knowledge were transformed to scores between 0 (no correct answers) and 1 (all answers correct).

With respect to Critical Literacy, only athletes who received anti-doping education could answer the questions (*n* = 689). Item one, pertaining to whether they felt confident to educate other athletes based on their own education, was answered by 534 athletes, 12.2% (*n* = 65) of whom did not feel confident. Regarding item two, pertaining to whether they felt confident to report doping athletes (whistle blow) (*n* = 460), 15.2% (*n* = 70) of the respondents did not feel confident. There was no difference on the scores for these items according to whether the athletes received information only or education (Chi square = 0.24, *p* = 0.62 and Chi square = 2.0, *p* = 0.16, respectively).

##### Marker Sensitivity to Detect Changes in DAL, Perceived Legitimacy, and Trust Based on the Doping Education Received

Details on the statistics are displayed in Supplementary Tables 3, 4. We found significant differences in almost all markers with respect to the level of anti-doping education received. In detail, the more anti-doping education that athletes received, the higher the levels of Functional and Interactive Literacy. There was no significant difference in Functional Literacy between the information and comprehensive education groups. However, compared to the no education group, the information only group exhibited significantly improved test performance. This also applied to all items for the Interactive Literacy stage. Likewise, the perceived legitimacy and trust increased with the level of knowledge, except for the item pertaining to rules being implemented globally and equally, the mean value of which was the lowest among all three legitimacy items. Scores for this item did not differ by education level. The IOC was the only federation for which education did not impact perceived trust on all items (i.e., member concerns and keeping promises). For all other items, education increased perceived trust, with comprehensive education being associated with the highest values.

### Results Of Phase Two

#### Step 1: Results of the Second Expert Meeting—Phase One Discussion

The aim of phase one was to develop an effective assessment tool with high content validity, in collaboration with internationally renowned experts in anti-doping, to fulfill the overall aim of the research project. In this phase, the focus was on the discriminatory power of the items, sensitivity of the markers to detect differences in doping prevention strategies, and determination of whether anything should be added to GRADE IT based on the feedback of the athletes and developments in doping prevention initiatives.

Based on the first expert meeting, four markers were included (distributed among the three levels of Functional, Interactive and Critical Literacy) based on their potential to denote code-compliant behavior: perceived knowledge, test knowledge (Functional Literacy), confidence in dealing with pressure (Interactive Literacy) and the confidence to whistle blow (Critical Literacy). The latter marker used a “True”/“False” format, so the results are not presented in Supplementary Tables 3, 4, but rather in the text. Two markers of legitimacy and trust were also included, as they were considered relevant to anti-doping education (Shelley et al., 2021) as well as compliance with anti-doping rules (Woolway et al., 2020). They might exert indirect effects on the final decision to be code-compliant, although this is speculative and was not tested as part of this research. Most of the items had satisfactory discriminatory power; only the item pertaining to confidence of being successful in one's chosen sport without doping had low discriminatory power, reflected in the generally high scores and low standard deviation. Thus, it can be inferred that all athletes were confident of success in their sport. As this item did not provide any additional information, it was omitted from GRADE IT.

Almost all of the included variables demonstrated the ability to detect differences according to anti-doping education; the answers differed significantly according to that variable, except for the two Critical Literacy items (asking athletes whether they felt confident in educating other athletes or reporting a doped athlete). One reason for this could be that the “no education” group was not analyzed (because this group did not receive the question), and the items were not sensitive enough to detect differences between athletes who received information only compared to those receiving comprehensive education. Another reason could be that the answer scale did not allow for nuanced answers.

In the discussion with the experts, we focused especially on the incongruence between athletes who reported receiving anti-doping education (either comprehensive education or information) despite residing in a country that does not provide any such education, as established by Gatterer et al. (2020). This positive incongruence [that is athletes who reported to have received comprehensive education or information but come from a country that does not provide any education or only information seems easy to explain as the study of Gatterer et al. (2020)] could be explained by such athletes receiving information or education from other sources, such as their federation, school, or club, where Gatterer et al. (2020) recorded only the prevention initiatives of NADOs. It is more challenging to explain why some athletes from countries offering information or comprehensive education indicated that they had not received such education, or only information (negative incongruence).

#### Step 2: Refining GRADE IT

Based on the discussions during the second expert meeting, four areas requiring changes were identified. First, we changed the wording for some questions based on the athletes' feedback and discussions with the experts during the second meeting. Additionally, we changed and added some items to improve specificity, as we felt that some items addressed two aspects concurrently. For example, we changed the initial wording of “After a positive doping test…” to “After being caught doping…,” because the correct answer here could also be to open the B-sample. Furthermore, for items pertaining to perceived trust, we replaced “judiciary” with “legal system,” as we suspected that not all athletes fully understood the meaning of the former word. Additionally, we changed “their members” (referring to federations) to “their people/athletes” to enhance precision (all changes are listed in Supplementary Table 2).

Second, the operationalization of Critical Literacy was evaluated. In alignment with the other two levels of DAL, we expected to see a difference in Critical Literacy between athletes with no education and those who have received education. However, this item was only presented to athletes who indicated that they had received education. Thus, the item was transformed into a filter question and also presented to athletes who had not received any education. Additionally, the answer scale was changed to a Likert format for consistency, and the items were revised to be more specific and cover a broader spectrum of Critical Literacy. In detail, we changed one item to “…know how to take action (reporting doping, whistleblowing)” and also added the item “… would report a doped teammate.”

Third, the issue of incongruence between the education reportedly received by the athletes and that actually provided by the NADOs warranted attention. We decided not to change GRADE IT in this regard, but rather to add an additional aim to phase two of the study. In detail, we aimed to determine whether athletes' levels of DAL, trust and legitimacy differed according to educational incongruence. The limitation of the classification of Gatterer et al. (2020), i.e., the inclusion only of anti-doping education provided by NADOs, is further discussed in the limitations section.

Fourth, we realized that we neglected to include the respective NADOs within the perceived trust questions, and therefore added them.

#### Step 3 Implementation of the Final Tool

##### Sample Description and Level of Education Received

In phase two, 1,255 athletes (27.4% of all athletes attending the events) fully completed the survey and were included in the data analyses; their socio-demographic characteristics are described in Table 3.

**Table 3:** Socio-demographic characteristics of the study cohort—phase two.

	***n***	**%**	
**Event**
Baku (Summer EYOF)	695	55.4	
Lausanne (Winter YOG)	560	44.6	
**Origin**
Europe	1.094	87.6	
Asia	83	6.2	
North America	37	3.0	
South America	13	1.0	
Oceania and Africa	23	1.8	
**Gender**
Female	624	49.6	
Male	523	41.7	
**Sport**
Individual	893	71.2	
Team	215	17.1	
**Member of RTP**	272	21.7	
**Gender**
Male	523	41.7	
Female	623	49.6	
	**Mean**	**SD**	**Min-Max**
**Age**	15.98	0.97	14-18

*% values do not all sum to 100% due to missing data*.

*Due to low participant numbers, Oceania and Africa were combined to ensure data protection; YOG, Youth Olympic Games; EYOV, European Youth Olympic Festival; RTP, Registered Testing Pool*.

**Figure 5:**
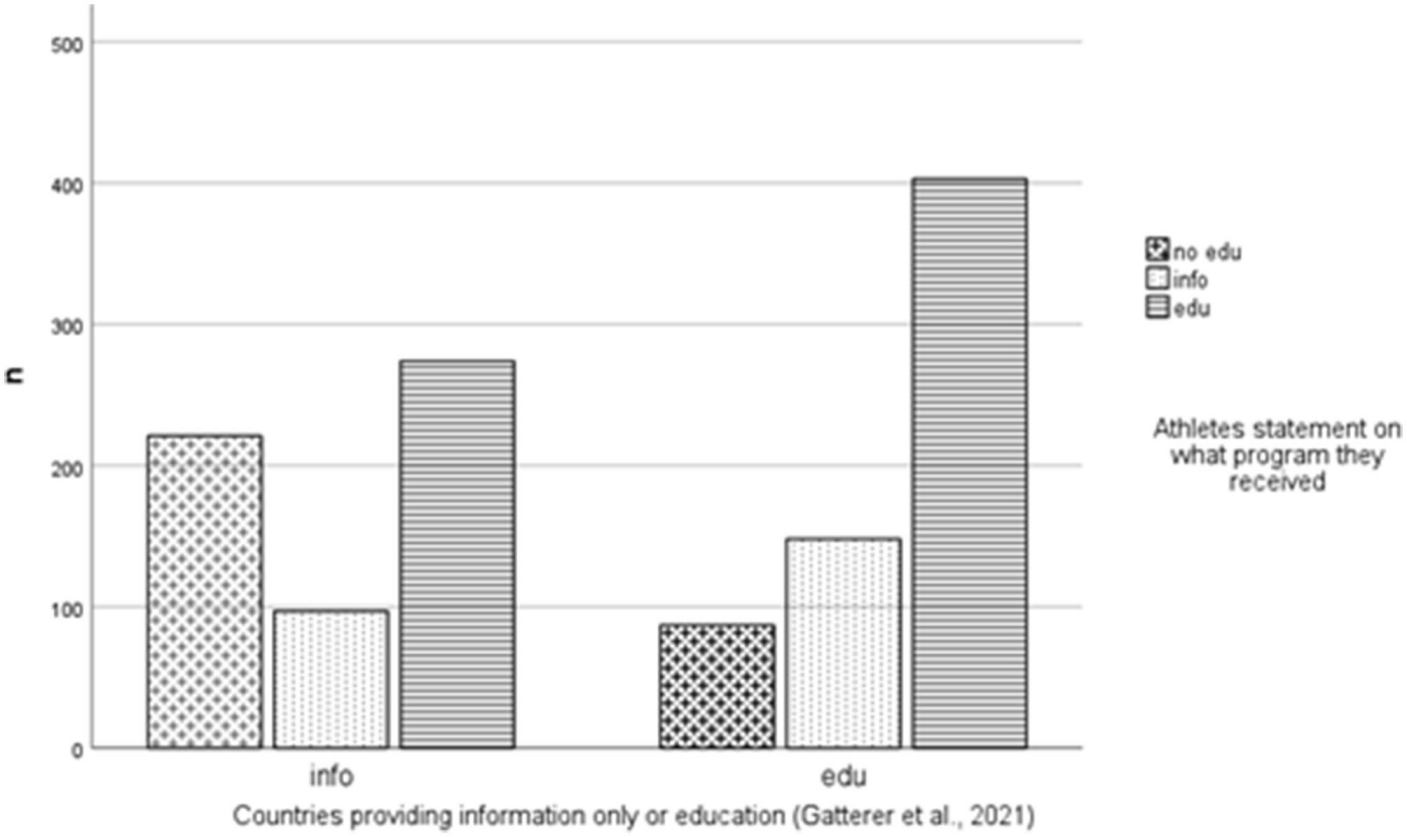
Distribution of perceived education of athletes from countries providing either information or education. Chi square 91.90, *p* < 0.001, Phi = 0.273.

Of the 1,255 elite youth athletes, 25.1% had never received anti-doping education, 19.8% received information and 54.3% received comprehensive education; 0.8% did not provide an answer. Based on the country classification of Gatterer et al. (2021), 47.4% of the athletes (*n* = 595) originated from countries whose NADOs provided information only, and 51.3% (*n* = 644) were from countries whose NADOs provided comprehensive education. For 1.3% of the respondents (*n* = 16), the country was not analyzed due to missing data. Of the 595 athletes from countries whose NADOs technically provide information only [as defined by Gatterer et al. (2020)], three were not included in further analysis because of missing data. Of the remaining 592 respondents, 37.3% (*n* = 221) indicated that they had never received education and 46.3% (*n* = 274) indicated that they had received comprehensive education (i.e., including role plays, for example). Of the 644 athletes from countries whose NADOs technically provide comprehensive education [as defined by Gatterer et al. (2020)], six were excluded from further analysis due to missing data. Of the remaining 638 respondents, 13.6% (*n* = 87) indicated that they had never received education and 23.2% (*n* = 148) reported only receiving information. Chi square analysis revealed significant educational incongruence (see Figure 5).

##### Impacts of Anti-doping Education on DAL, Perceived Trust, and Legitimacy

All items and scales, including the modified and newly added ones, showed similarly high discriminatory power and similar difficulty (for details, refer to Supplementary Tables 5, 6). We could even validate the Functional Literacy scale as there was an association between the level of perceived knowledge and “don't know answers” of the test knowledge questions. In detail, as expected, athletes who answered don't know had a significantly lower level of perceived knowledge compared to those who correctly or incorrectly answered in all items expect three (for these three, it was only significantly lower compared to athletes who gave the correct answer). Therefore, we decided to further analyze the data to determine whether we can find support for our expectation that anti-doping education impacts on DAL and, even if not a direct education goal, influences the perceived legitimacy of anti-doping and perceived trust of organizations.

**Figure 6:**
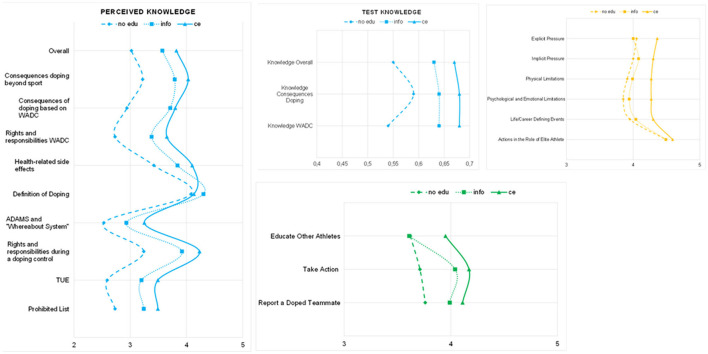
The DAL stages. Functional Literacy: blue; Minimum–Maximum Perceived Knowledge: 1–5; Minimum–Maximum Test Knowledge: 0–1 → figures are scaled to provide more detailed information; PK, perceived knowledge; WADC, World Anti-Doping Code; TUE, Therapeutic Use Exemption; ce, comprehensive education. All changes were significant. For details on *p*-values and *post-hoc* tests, refer to Supplementary Table 5. Interactive Literacy: orange; Minimum–Maximum: 1–5; → figure is scaled to provide more detailed information; ce, comprehensive education. All changes were significant except for “Actions in role as an elite athlete.” For details on *p*-values and *post-hoc* tests, refer to Supplementary Table 5. Critical Literacy: green; Minimum–Maximum: 1–5; → figure is scaled for more detailed information; ce, comprehensive education. All changes were significant. For details on *p*-values and *post-hoc* tests, refer to Supplementary Table 5.

**Figure 7:**
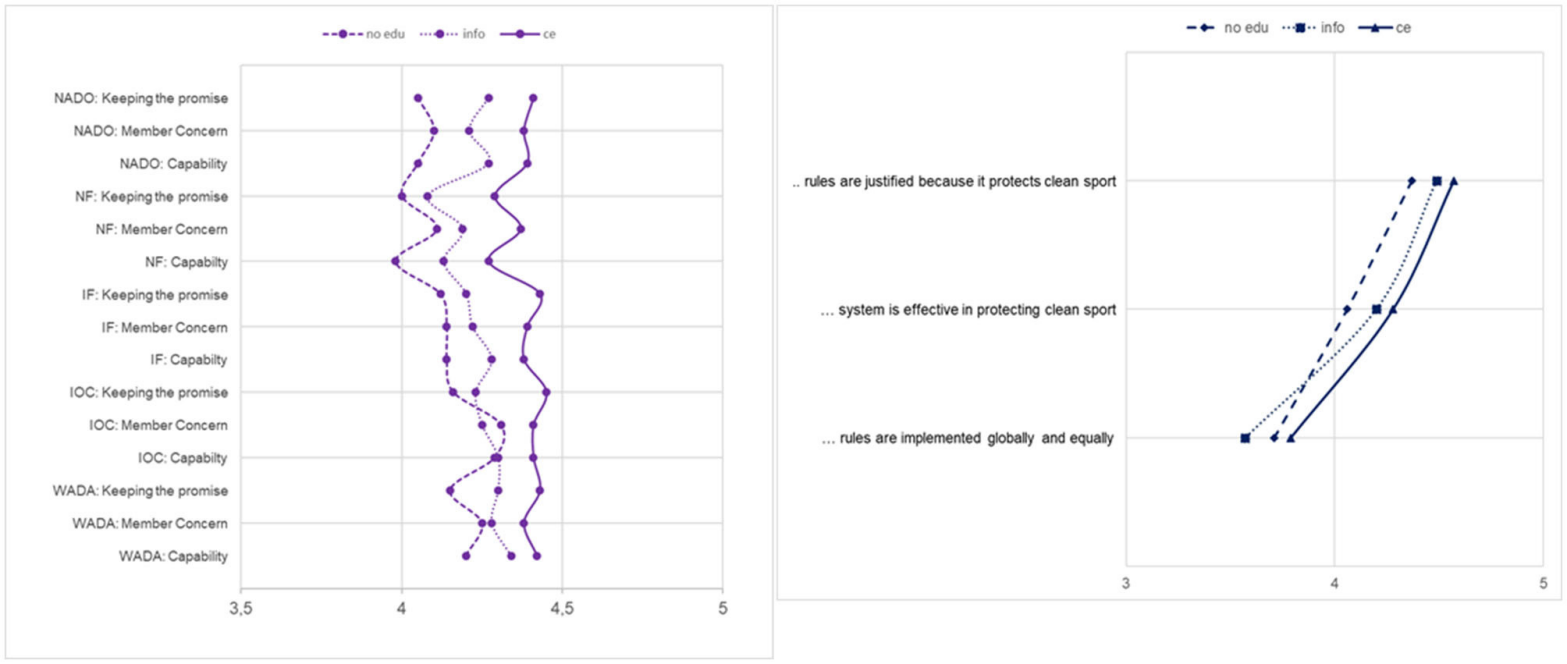
Differences in perceived trust and legitimacy according to the level of education received. Trust: Minimum–Maximum: 1–5; → figure is scaled to provide more detailed information; ce, comprehensive education. All changes were significant, except for “WADA: member concern” and “IOC: capability.” For details on *p*-values and *post-hoc* tests, refer to Supplementary Table 5. Legitimacy: Minimum–Maximum: 1–5; → figure is scaled to provide more detailed information; ce, comprehensive education. All changes were significant, except for “rules are implemented equally and globally.” For details on *p*-values and *post-hoc* tests, refer to Supplementary Table 5. NADO, National Anti-Doping Organization; NF, National Sport Federation; IF, International Sport Federation; IOC, The International Olympic Committee; WADA, World Anti-Doping Agency.

As expected, the developed tool was sufficiently sensitive to detect differences in all DAL stages, as well as perceived anti-doping legitimacy and trust. Athletes with a comprehensive education had the highest scores for Functional, Interactive and Critical Literacy, as well as perceived trust and anti-doping legitimacy. The only exception was the “fair process” component of procedural legitimacy, for which the scores were uniformly low (compared to the other components of legitimacy) regardless of education level. Also, for parts of Functional Literacy, the provision of information made a difference (relative to no education). With respect to Interactive and Critical Literacy, information alone does not seem to be sufficient, as the scores did not differ significantly for those athletes compared to the no education group. For details, refer to Figures 6, 7 and Supplementary Tables 5, 6.

##### Impact of Educational Incongruence

As indicated above, an additional (“*post-hoc*”) research question arose from the discussion that took place after phase one, pertaining to the educational incongruence defined previously. For these analyses, only athletes from countries whose NADO provided any kind of education (*n* = 1,239), as defined by Gatterer et al. (2020), were included, as we did not have sufficient information for the remaining countries. Both positive and negative incongruence had significant effects on DAL. In detail, all DAL stage scores were highest if athletes receiving comprehensive education, and in most cases, information improved the scores compared to no education. However, the highest scores for perceived knowledge (Functional Literacy), some aspects of Interactive Literacy (i.e., confident in the role of elite athlete, and in dealing with career/life changing events and physical limitations), and Critical Literacy were achieved by athletes with congruence in terms of the reported and actually received education; athletes who received comprehensive education from countries who were classified as providing it scored highest. The results are graphically displayed in Supplementary Figures 1, 2, and detailed statistical information is provided in Supplementary Tables 7, 8.

### Phase Two Discussion

The aim of phase two was to use the assessment tool refined in phase one to determine whether anti-doping education makes a difference to Functional, Interactive and Critical Literacy, as well as to perceived trust and legitimacy. Additionally, the impact of educational incongruence was further analyzed. In sum, the extent of education received (no education, information only, comprehensive education) had an impact on almost all markers assessed; as expected, athletes who received comprehensive education scored highest for all markers. In line, a recent systematic review on doping prevention measures targeting young age groups also concluded that programs that actively engage participants are considered to be better than lecture-based knowledge transfer (Pöppel, 2021). Regarding the DAL components, educational incongruence seemed to have a negative effect, especially for Critical Literacy. Even though the ISE (World Anti-Doping Agency, 2021b) was not in effect when this study was planned and implemented, the results fit well with most parts of the ISE, as we outline below.

### Impact of Education on the Doping Awareness Literacy Stages

Information-only approaches had a significant effect on Functional Literacy and one aspect of Critical Literacy. However, according to our results, comprehensive education was crucial for both Interactive Literacy and Critical Literacy.

Elements of Functional Literacy included perceived and actual (test) knowledge about anti-doping rules, and athletes' roles and responsibilities under the WADC. These elements can be classified according to the ISE's two components of awareness-raising and information provision (World Anti-Doping Agency, 2021b), and represent information according to the classification of Gatterer et al. (2020). As expected, the scores on all items pertaining to these variables were significantly higher for athletes who received information at least. Interestingly, the level of perceived knowledge even showed a significant difference between the group of athletes who received information only and those who received comprehensive education. Whereas the test knowledge increase associated with information provision was not further increased by comprehensive education, perceived knowledge can be further increased with comprehensive education. In the context of DAL, perceived knowledge might be associated with Interactive Literacy, as this refers to the ability to apply knowledge. It could be hypothesized that the level of perceived knowledge of the athlete is more closely associated with acting on such knowledge compared to test knowledge. In line with this idea, comprehensive education was more important for Interactive Literacy and Critical Literacy. Overall, the results suggest that information-based prevention is sufficient to ensure a high level of Functional Literacy, which is an important component of DAL. In this context, the fact that most global anti-doping organizations offer information-based education is advantageous (Gatterer et al., 2020). However, as stated in the Introduction section, Functional Literacy alone is not sufficient to increase self-efficacy with respect to making informed decisions about doping.

The second important concept, of Interactive Literacy, refers to the ability to apply learned knowledge and skills in complex situations, to make the right decision with respect to PIED use. This stage also demands understanding of the situational context and sources of resilience (Petróczi et al., 2021b). Interactive Literacy can be related to the anti-doping education of the ISE, which is intended to empower athletes to make informed decisions regarding clean sport (World Anti-Doping Agency, 2021b). Interactive Literacy can be operationalized as the confidence to deal with pressure based on the education received (if any). The results show that information-only approaches (aligned to the ISE components of information provision and awareness-raising; World Anti-Doping Agency, 2021b) do not affect the level of Interactive Literacy, but only the provision of comprehensive education (aligned to the ISE components of value-based education and anti-doping education; World Anti-Doping Agency, 2021b) seems to have an effect. As with Functional Literacy, the athletes who received comprehensive education displayed the highest scores for Interactive Literacy. This points to the first gap that needs to be addressed by all organizations entrusted with anti-doping education, where research has shown that comprehensive education is provided by relatively few international ADOs (Gatterer et al., 2020), even though it seems crucial to ensure a high level of Interactive Literacy, which in turn leads to Critical Literacy.

Regarding Critical Literacy, defined as the ability to think beyond the “self” (Petróczi et al., 2021b), the results are more diverse. Information-only approaches do not seem to increase the confidence of athletes to educate others about anti-doping, but appear sufficient for increasing athletes' confidence to report on doping (whistle-blowing). The confidence to whistle blow is further increased by comprehensive education. The outcomes of Critical Literacy can be assigned to the values-based component of the ISE (World Anti-Doping Agency, 2021b); similar to anti-doping education, it has not yet been implemented widely in doping prevention measures (Gatterer et al., 2020). Critical Literacy is especially important, as we expect it to be critical not only for making the right decision in the context of doping itself, but also in terms of being an ambassador for a clean sport identity, which has been shown to be a strong protective factor against doping (Petróczi et al., 2021b).

Finally, we would like to further discuss the point made in the Introduction that doping prevention should be rephrased to anti-doping education. The mean scores for all of the literacy stages were above the midpoint of the scale (i.e., > 3.5 on a Likert scale), even for athletes who did not receive any education, which might be by chance due to the True/False format of the tool. Yet, it might be argued that the role of education is not to “prevent” something, as the athletes showed high levels of literacy from the outset, but rather to augment the “good” that is already there. This is in line with the belief that most athletes want to compete in a clean sport environment, show clean sport behavior and comply with anti-doping rules. This supports the new perspective of focusing on why athletes do not dope, and strengthening protective factors that help them making the right choices. It must be noted that the study cohort comprised adolescent elite athletes (aged between 14 and 19 years), where scores might be affected by the age and experience of the athletes. As outlined in the Introduction, doping can be understood as a coping strategy (Petróczi and Aidman, 2008) for dealing with multiple risks arising from the interaction between the environment and the individual (Petróczi, 2013; Petróczi et al., 2017). In this respect, age is important because perceived risks change with age. Likewise, given that elite sport is an exceptional and sometimes risky environment, in which drug use behavior is influenced by specific social and cultural factors and their complex interactions and interdependencies (at different levels in the sport figuration), athletes' reactions (and pressures) may change when facing new working conditions, for example (Overbye, 2018). Thus, it seems likely that perceptions of risks and pressures may change with age over the course of a sporting career. While it seems likely that functional literacy would have even higher scores among adult athletes who regularly receive anti-doping information and education, further research is needed to assess whether scores for interactional literacy are affected by age based on the arguments delineated above. Even though our tool was developed with youth athletes, we are confident in its applicability to samples of older athletes.

### Impact of Education on Trust and Legitimacy

The overall trust scores (combined scores for the items on capability to fulfill the role, taking care of members and keeping promises) were between 3 and 4 on the Likert scale, and clearly showed that only comprehensive education significantly increases the level of trust of federations (IOC, WADA, NADO, International and National Federation), as perceived by athletes. If trust plays a role in literacy and compliance in anti-doping (Dreiskämper et al., 2016) as important as that which it plays in health science, there is a need for comprehensive anti-doping education. Similar results (and conclusions) were found for legitimacy; however, this concept needs to be discussed in more detail. As defined by Tyler (2006), perceived legitimacy requires that a system operate in a proper, just, and appropriate manner. Following Woolway et al. (2020), the current research distinguished normative legitimacy from two types of procedural legitimacy (fair outcome and fair process), as outlined in the methods section and used in other studies [e.g., a qualitative study by Qvarfordt et al. (2021); for details, refer to the Supplementary Material 2].

Athletes who received comprehensive education scored significantly higher on normative legitimacy (system perceived to be appropriate) components, and on one component of procedural legitimacy (system being perceived to be proper), compared to those only receiving information-based education or no education. These findings are especially important in terms of the concept of appropriateness, as there is evidence that the effectiveness of the system is still considered weak (Woolway et al., 2020). Athletes around the world agreed that anti-doping organizations are doing the right things (i.e., proper) but not always in the right way (i.e., [in]appropriate), which is highly relevant to compliance with the system according to the athletes themselves. Woolway et al. (2020) suggested that promoting the results of anti-doping authorities might improve perceived appropriateness. The findings of the current study highlight the importance of comprehensive education (including value-based education), because information-based education does not appear to significantly improve perceptions of the system as proper or appropriate compared to no education.

The second component of procedural legitimacy (perceiving the system as just), however, was not affected by the kind of education received. Scores were lowest for this component, and were not significantly different among the groups of athletes who received no education, information, or comprehensive education. The lower scores on this component compared to the other two were expected, given that previous research showed that most elite athletes have low trust in the equal implementation of doping controls globally (Bourdon et al., 2014; Overbye and Wagner, 2014; Efverström et al., 2016; Overbye, 2016). However, the average score (i.e., >3.5 on a Likert scale) cannot be considered low. It seems likely that the relatively high levels of trust in our cohort might be related to their young age; samples with older and more experienced elite athletes may have lower scores (i.e., higher levels of distrust regarding equal and fair implementation of anti-doping measures globally). For example, studies showed that trust in specific parts of the anti-doping system were higher among younger athletes, but decreased with age (Overbye, 2016). Moreover, athletes with personal experience of specific anti-doping measures/procedures showed lower trust in their functioning (Overbye and Wagner, 2013, 2014). Athletes' trust may change during their sporting career; in particular, negative experiences (e.g., of system errors) can lead to distrust in the anti-doping system (Overbye, 2016). Also, the review of Woolway et al. (2020) outlines how athletes generally support anti-doping measures because they agree that they are necessary. However, even though included athletes in the study of Woolway et al. (2020) articulated high levels of trust in their own anti-doping system, they also expressed concerns about the global fairness of the process and its outcomes. Linked to the experience of athletes, Woolway et al. (2020) showed that athletes only appreciate the issue of global fairness once they start competing internationally and thus accrue first-hand experience with how anti-doping rules are followed in countries other than their own. Woolway et al. (2020) suggest that low trust in the implementation of anti-doping measures might also arise due to a lack of knowledge about the anti-doping activities of different organizations and countries. Information about anti-doping activities is not part of most education-based prevention measures (Gatterer et al., 2020), which might help to explain why the scores for the just concept (perceived procedural legitimacy) did not differ by education level in this study. Importantly, knowledge about the implementation of anti-doping activities should correspond to reality (i.e., what is actually offered), which underscores the importance of improving compliance with and implementation of doping rules across the world. Studies illustrating that unequal or inappropriate implementation can also undermine non-doping athletes' trust in anti-doping further support this (Overbye, 2016; Shelley et al., 2021). The latter (as mentioned earlier) may ultimately be associated with rule compliance and acting on the DAL (Woolway et al., 2020). We hypothesize that DAL with high levels of perceived trust and legitimacy would not only improve athletes' ability to make informed decisions, but also to *want* to make them, as they would perceive the anti-doping system as legitimate, and the organizations entrusted with it as trustworthy.

### Impact of Educational Incongruence on Doping Awareness Literacy Stages, Perceived Trust, and Legitimacy

The results of both phase one and two indicated significant incongruence between what athletes should have received in terms of anti-doping education and what they in fact received. On the one hand, there were athletes who indicated receiving comprehensive education even though they resided in countries in which NADOs only offered information-based education, according to Gatterer et al. (2020). An explanation for this positive incongruence may simply be that the athletes received education from other providers, as already discussed in phase one. However, the study also identified negative incongruence: athletes not receiving education despite residing in countries whose NADOs provide it. This latter finding merits attention [see also Gatterer et al. (2021)], especially because the results of phase two clearly illustrate that comprehensive education had significant effects on DAL. As discussed above, scores for all DAL stages were highest among athletes who received comprehensive education, but information-based education also improved scores compared to no education. Regarding translation of this finding into practice, it might not matter what kind of education the responsible organizations provide to their athletes (with comprehensive education leading to high DAL, trust, and legitimacy) but, regarding DAL, whether the athletes are aware that they received this education is important. None of these points were applicable to the concepts of trust and legitimacy.

## Limitations And Future Research

This research was not free of limitations. The first set of limitation addresses the tool itself, whereas the second set addresses more generic limitations. Even though the development of the survey followed a strict and systematic approach, final items were chosen based on consensus of the research group. Bias based on wording and/or the fact of including some and not including other items that as well might have reflected the latent construct cannot be fully excluded but were mitigated against by striving for consensus in the research group and experts. Furthermore, the knowledge questions used to supplement the perceived and test knowledge that underpons Functional Literacy covered knowledge about the strict liability principle, rights and responsibilities during doping controls, possible ADRVs and the whereabout system. These questions were thus driven by anti-doping rule violation (as it was the time with the 2015 Code) and did not cover all possible knowledge areas, as for example knowledge of the prohibited list. We decided to opt for a more generic knowledge that is applicable to all athletes. Because drug specific knowledge is sport specific, no athlete (or athlete support personnel for that matter) can reasonably be expected to know about all drug classes in the Prohibited List. Even though we believe that the items chosen reflect Functional Literacy and we could show that education impacted this specific knowledge, GRADE-IT could and should be validated with a different set of knowledge questions. Further facilitating this is the need that the current set of knowledge questions must be updated for the World Anti-Doping Code that came into effect in 2021, because ADRVs and athletes' roles and responsibilities changed. Another limitation that is worth noting is the stem of the questions in regard to perceived knowledge. One could argue that “how well you know” and “how well do you feel informed about” are not the same as one could feel well informed (the information is out there or has been provided, or they have access) but one is not particularly knowledgeable. Thus, this question might be formulated too imprecise and future research using the tool might want to consider changing the wording. To be consistent with the literacy approach, that reflects the capability to solve a situation rather than knowing an answer to every question, we would suggest to only use “how well do you feel informed about.”

In terms of more generic bias, we were only able to compare cohorts of athletes with different anti-doping education experiences to demonstrate the utility of our tool as a cross-sectional retrospective design was used. Thus, we cannot confirm that anti-doping education causes or changes the DAL stages; a prospective cohort study is needed to validate the tool. Additionally, although GRADE IT is available in 23 languages, a language bias cannot be ruled out, where some athletes might have misinterpreted items due to a language barrier. Furthermore, as doping, trust and legitimacy are sensitive issues, socially desirable responding cannot be excluded. Also, the mean values may have been higher than what would be expected in a sample of older athletes, and we assume that most of the athletes were not doping (as discussed earlier). Additionally, there might have been a selection bias, whereby the participating athletes may have had a generally positive attitude toward anti-doping education and perceived the system as trustworthy and legitimate. Importantly, even though the mean values for most of the constructs were high, they still differed among the assessed groups and ceiling effects seem unlikely. In terms of the representativeness of the sample, 23.5% of all athletes who attended the four events participated in the study, i.e., we did not receive information from every athlete. As the survey was not sent out to all athletes, we tried to reach as many as possible through various channels; consequently, we cannot confirm the true response rate. It was not feasible to collect data from all attending athletes, because some of them did not have contact with us, others did not have the time, and still others did not spend their free time in the communal spaces where we provided the tablets. Finally, some of the athletes did not want to participate because of the sensitivity of the topic. We tried to counter these difficulties by providing the link through as many channels as possible (as outlined in the methods section), and by ensuring complete anonymity. Unfortunately, it is not feasible to evaluate whether or not the missing values were at random. As stated earlier, the positive educational incongruence was most likely due to Gatterer et al. (2020) only analyzing NADOs, even though there are other organizations that provide anti-doping education. However, as we showed that comprehensive education is important for DAL, as well as for trust and legitimacy, we expect that the inclusion of other organizations would not alter this finding, as these organizations also mostly provide information-based education (Hurst et al., 2020). With respect to the negative incongruence, it is possible that some athletes who indicated that they had not received any kind of education were not aware that they had in fact received such education. Finally, the label of “comprehensive education” included all education initiatives going beyond providing only information on what is prohibited or listed as rules and responsibilities under the WADC. We did not further differentiate between the content (e.g., checking whether values-based education was part of it). The self-report nature of the data on the education received by the athletes is a further limitation.

We believe that developing a valid and reliable instrument for evaluation is an iterative process, often with series of empirical testing not just by the developers but the broader research community, and in many cases with feedback from practical implementation. What we present here is a starting point for this process, not the ultimate product. Thus, future research should apply our tool in a setting in which the education provided is controlled and can be classified more precisely. This would provide additional important information on the role of values-based education in the development of DAL, for example.

## Practical Implications

The two main findings of this study were as follows: (a) we were able to develop a reliable assessment tool, GRADE IT, which includes relevant markers with sufficient sensitivity to detect changes in DAL stages, perceived trust and legitimacy; and (b) differences were identified in almost all these parameters according to the anti-doping received. The focus needs to be on the importance of comprehensive education because, as the highest level of anti-doping education (also requested by the ISE), it has the greatest effects on Functional Literacy and Critical Literacy, as well as on trust and aspects of perceived legitimacy. Unfortunately, research has shown that comprehensive education is the least well-implemented type of education by NADOs worldwide, even though some of them erroneously believe that they do in fact provide it (Gatterer et al., 2020). Additionally, considering that elite athletes will always be operating in a high-pressure environment that can itself be considered a risk factor for doping (Whitaker et al., 2017; Gatterer et al., 2019), a high level of DAL in all aspects, as well as clean sport culture, might promote resilience to pressure. In the context of the finding that comprehensive education impacts positively on Critical Literacy, implementation of the ISE and all four components [values-based education (awareness-raising, information and anti-doping education)] is expected to lead to closer alignment between education content and intended outcomes, which is currently lacking (Woolf, 2020). Additionally, we call for additional research on intervention activities provided by organizations other than NADOs.

A further practical implication of this research pertains to the need to enforce the awareness-raising ISE component (World Anti-Doping Agency, 2021b), not only in the context of doping-related issues but also in the context of why athletes must take specific anti-doping classes. This seems important because negative educational incongruence significantly impacted DAL. Additionally, promoting the results of anti-doping authorities, and knowledge and awareness of the anti-doping and education of different organizations and countries, might also have a direct effect on perceived legitimacy (i.e., enhance perceptions of the system as appropriate and just). These are particularly important aspects of perceived legitimacy, because it is hypothesized that these are associated with compliance with the system (Woolway et al., 2020).

**Figure 8:**
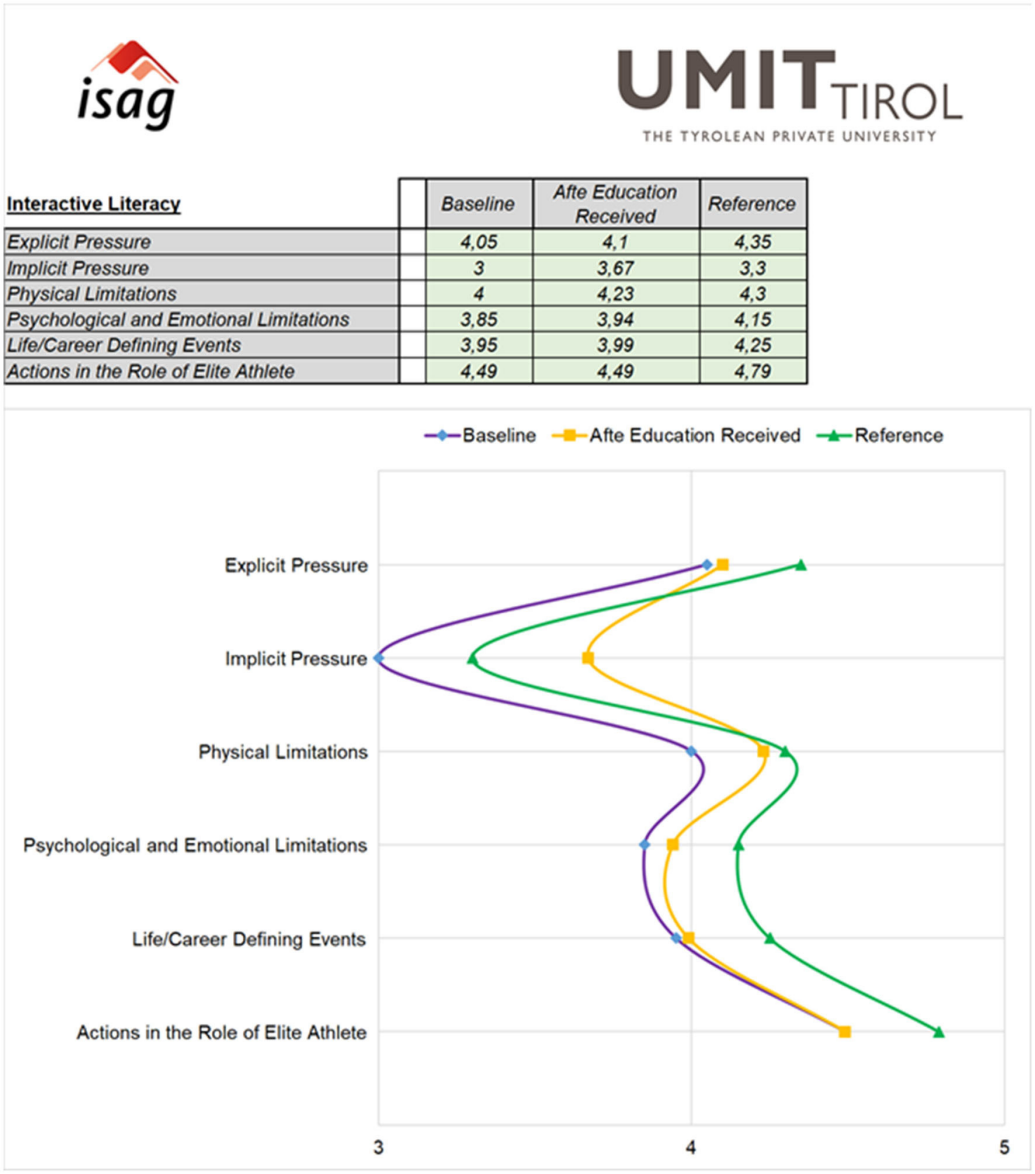
Illustrative example of profile graph (excel template) for organizations.

Finally, we invite researchers to use GRADE IT (it is available online in 23 languages, at osf.io/vjtrz) to collect additional data to: (a) determine whether our results can be replicated in other samples, (b) assess if other variables such as culture, sport and level of competition are effect modifiers, (c) build a database to identify if changes in doping prevention policies have a delayed effect on the concepts of DAL, perceived trust and legitimacy, and (d) assess if these concepts are associated with “making the right decision” (this is likely to be the most challenging task). WADA's ISE requests organizations delivering anti-doping education to develop evaluation strategies to assess its effectiveness. To support this, we welcome organizations to use GRADE IT as evaluation tool. There is an Excel Template provided at osf.io/vjtrz that will allow the organization to enter its own data to receive representative figures that are similar to the ones presented in this paper. To make full use of the tool and the profile map (see Figure 8 for an illustrative example), organizations using the GRADE-IT should collect baseline data first in order to have the first line in the figure representing mean values of the current levels of DAL, perceived trust and legitimacy. Then, to add a second line where they asses the mean values achieved after receiving anti-doping education. The third line serves as the reference line which is set by the organization as the minimum target. With these profile graphs, representing group means instead of individual assessments, organizations will have a valuable visual tool to evaluate their anti-doping program.

## Conclusion

In conclusion, there is a need for performance-based evaluations of anti-doping education mandated by the International Standard of Education. With GRADE IT, we offer an evaluation tool that is available in 23 languages and focuses on athletes' capabilities to make the right decision as it pertains to clean sport behavior context. We showed that the tool worked well when applied to a sample of elite adolescent athletes. Before implementation of the tool, further work is warranted (i.e., validation in an adult athlete population, and application in different settings where anti-doping education is not self-reported but established independently). Future research could focus on applying the tool longitudinally to examine whether changes in doping prevention policies have a delayed effect on DAL stages, perceived trust, and legitimacy.

## Data Availability Statement

The raw data supporting the conclusions of this article will be made available by the authors, without undue reservation.

## Ethics Statement

The studies involving human participants were reviewed and approved by Research Committee for Scientific Ethical Questions (RCSEQ), UMIT - Private University for Health Sciences, Medical Informatics and Technology. Written informed consent to participate in this study was provided by the participants' legal guardian/next of kin.

## Author Contributions

CB and AP: conceptualization and supervision. CB, KG, MO, WS, BS, and AP: methodology and writing—review and editing. CB and KG: formal analysis and investigation. KG: data curation and project administration. CB, KG, and AP: writing—original draft preparation. CB: funding acquisition. All authors have read and agreed to the published version of the manuscript.

## Funding

This study received funding from the Anti-Doping Research Grant of the International Olympic Committee (IOC).

## Conflict of Interest

WS was employed by Tirol Kliniken GmbH. The remaining authors declare that the research was conducted in the absence of any commercial or financial relationships that could be construed as a potential conflict of interest.

## Publisher's Note

All claims expressed in this article are solely those of the authors and do not necessarily represent those of their affiliated organizations, or those of the publisher, the editors and the reviewers. Any product that may be evaluated in this article, or claim that may be made by its manufacturer, is not guaranteed or endorsed by the publisher.
