# Supplementary material for: GRADE IT—A Literacy-Based Assessment Tool for Generating Research-Based Assessment Data to Evidence the ImpacT of Anti-doping Education via Athletes' Capability to Make the Right Decision
Source: Front Sports Act Living. 2022 Mar 15;4:842192. doi: 10.3389/fspor.2022.842192 (PMC8965155; doi:10.3389/fspor.2022.842192)
Supplement: Supplementary file 1 [file Data_Sheet_1.docx]

**Supplemental Material**

**Table S1**: *Initial Assessment Tool – Phase One*

| Have you ever received anti-doping education (e.g. lecture, info event etc.)? | | | | | | | | | | | | | | | | | | |
| --- | --- | --- | --- | --- | --- | --- | --- | --- | --- | --- | --- | --- | --- | --- | --- | --- | --- | --- |
| □ Yes  □ No | | | | | | | | | | | | | | | | | | |
| What were the contents of the education? (Multiple responses possible) | | | | | | | | | | | | | | | | | | |
|  | | | | | | | | | | | | | | | | | | |
| □ | Information about prohibited substances/methods | | | | | | | | | | | | | | | | | |
| □ | Information about anti-doping organizations and their responsibilities | | | | | | | | | | | | | | | | | |
| □ | Contents of the World Anti-Doping Code 2015 | | | | | | | | | | | | | | | | | |
| □ | Role plays (e.g. dilemma situations) | | | | | | | | | | | | | | | | | |
| □ | Online programs/apps | | | | | | | | | | | | | | | | | |
| □ | Discussions | | | | | | | | | | | | | | | | | |
| □ | Others: _________________________ | | | | | | | | | | | | | | | | | |
| Based on the doping-prevention education I received^[[1]](#footnote-1)^, I am confident that I can deal with the following situations without using prohibited substances and/or methods:  (1 = not confident at all - 5 = very confident) [SELF-EFFICACY – INTERACTIVE LITERACY] | | | | | | 1 | | 2 | | 3 | | 4 | | 5 | | | Cannot assess |  |
| … explicit pressure from my environment (e.g. coaches, peers, sponsors, federation, club) | | | | | |  | |  | |  | |  | |  | | |  |  |
| … physical limitations (e.g. injuries, illness, fatigue, overtraining) | | | | | |  | |  | |  | |  | |  | | |  |  |
| … psychological and emotional limitations (e.g. perceived lack of progress, perceived lack of trust in my own performance) | | | | | |  | |  | |  | |  | |  | | |  |  |
| … life-/career-defining events (moving away from home, junior to big league) | | | | | |  | |  | |  | |  | |  | | |  |  |
| … be successful in my sport while staying clean [SELF-CONFIDENCE] | | | | | |  | |  | |  | |  | |  | | |  |  |
| Based on the doping-prevention education I received, I am confident that I can … [SELF-EFFICACY – CRITICAL LITERACY] | | | | | Yes | | | | | No | | | | Cannot assess | | | |  |
| … educate other athletes | | | | |  | | | | |  | | | |  | | | |  |
| … report doped athletes (“whistleblowing”) | | | | |  | | | | |  | | | |  | | | |  |
| On a scale from 1 – 5 (not at all – very well), how well do you know or feel informed about… [PERCEIVED KNOWLEDGE – FUNCTIONAL LITERACY] | | | | 1 | | | 2 | | 3 | | 4 | | 5 | | | Cannot assess | |  |
| … the prohibited list of the World Anti-Doping Agency of 2018 | | | |  | | |  | |  | |  | |  | | |  | |  |
| … therapeutic use exemptions | | | |  | | |  | |  | |  | |  | | |  | |  |
| ... your rights and responsibilities during a doping control | | | |  | | |  | |  | |  | |  | | |  | |  |
| ... ADAMS and the "Whereabout System" | | | |  | | |  | |  | |  | |  | | |  | |  |
| … the definition of doping | | | |  | | |  | |  | |  | |  | | |  | |  |
| … the health-related side-effects of doping substances and methods | | | |  | | |  | |  | |  | |  | | |  | |  |
| … your rights and responsibilities under the World Anti-Doping Code 2015 | | | |  | | |  | |  | |  | |  | | |  | |  |
| ... the consequences of doping based on the World Anti-Doping Code 2015 | | | |  | | |  | |  | |  | |  | | |  | |  |
| ... the consequences of doping beyond a ban from sport (e.g. criminal law, loss of sponsoring) | | | |  | | |  | |  | |  | |  | | |  | |  |
| According to the World Anti-Doping Code 2015… [KNOWLEDGE] | | | True | | | | | | False | | | | Do not know | | | | |  |
| … you are not allowed to use or possess any prohibited substances and/or methods without a valid reason [ADRV] | | |  | | | | | |  | | | |  | | | | |  |
| … you can be banned from sport if you violate the anti-doping regulations of the Code [SANCTION] | | |  | | | | | |  | | | |  | | | | |  |
| … prohibited substances and/or methods are allowed after consultation with a physician [TUE] | | |  | | | | | |  | | | |  | | | | |  |
| … you are allowed to take any medication if you are ill as long as they are prescribed by a physician [TUE] | | |  | | | | | |  | | | |  | | | | |  |
| … you are allowed to take any medication if you are ill [TUE] | | |  | | | | | |  | | | |  | | | | |  |
| … you have to actively participate during doping controls [ADRV/RESPONSIBILITY] | | |  | | | | | |  | | | |  | | | | |  |
| … you are not allowed to refuse a doping control [ADRV/RESPONSIBILITY] | | |  | | | | | |  | | | |  | | | | |  |
| … if you are part of a testing pool, you have to provide detailed information about your whereabouts (training, competition) [ARDV/RESPONSIBILITY] | | |  | | | | | |  | | | |  | | | | |  |
| … the term “doping” also includes possession, trading and trafficking of prohibited substances and/or methods [ADRV] | | |  | | | | | |  | | | |  | | | | |  |
| … you alone are responsible for any substance within your body [STRICT LIABILITY] | | |  | | | | | |  | | | |  | | | | |  |
| After a positive doping test… [SANCTION] | | | True | | | | | | False | | | | Do not know | | | | |  |
| … you can be banned from your sport | | |  | | | | | |  | | | |  | | | | |  |
| … you can be banned from participating in any sport | | |  | | | | | |  | | | |  | | | | |  |
| … there might be additional consequences next to a ban from sports (i.e. legal consequences) | | |  | | | | | |  | | | |  | | | | |  |
| I trust that the following organizations are capable of fulfilling their responsibilities  (1 = not at all – 5 = very much) [TRUST IN ABILITY or CAPABILITY] | | | | 1 | | | 2 | | 3 | | 4 | | 5 | | | Cannot assess | |  |
| … the judiciary | | | |  | | |  | |  | |  | |  | | |  | |  |
| … the World Anti-Doping Agency (WADA) | | | |  | | |  | |  | |  | |  | | |  | |  |
| … the International Olympic Committee (IOC) | | | |  | | |  | |  | |  | |  | | |  | |  |
| … my national sports federation | | | |  | | |  | |  | |  | |  | | |  | |  |
| … my international sports federation | | | |  | | |  | |  | |  | |  | | |  | |  |
| I trust that the following organizations are concerned about their members  (1 = not at all – 5 = very much) [TRUST IN BENEVOLENCE] | | | | 1 | | | 2 | | 3 | | 4 | | 5 | | | Cannot assess | |  |
| … the judiciary | | | |  | | |  | |  | |  | |  | | |  | |  |
| … the World Anti-Doping Agency (WADA) | | | |  | | |  | |  | |  | |  | | |  | |  |
| … the International Olympic Committee (IOC) | | | |  | | |  | |  | |  | |  | | |  | |  |
| … my national sports federation | | | |  | | |  | |  | |  | |  | | |  | |  |
| … my international sports federation | | | |  | | |  | |  | |  | |  | | |  | |  |
| I trust that the following organizations keep their promises  (1 = not at all – 5 = very much) [TRUST IN INTEGRITY] | | | | 1 | | | 2 | | 3 | | 4 | | 5 | | | Cannot assess | |  |
| … the judiciary | | | |  | | |  | |  | |  | |  | | |  | |  |
| … the World Anti-Doping Agency (WADA) | | | |  | | |  | |  | |  | |  | | |  | |  |
| … the International Olympic Committee (IOC) | | | |  | | |  | |  | |  | |  | | |  | |  |
| … my national sports federation | | | |  | | |  | |  | |  | |  | | |  | |  |
| … my international sports federation | | | |  | | |  | |  | |  | |  | | |  | |  |
| Please indicate the level of agreement with the following statements:  (1 = strongly disagree – 5 = strongly agree) [LEGITIMACY PERCEPTION] | | | | 1 | | | 2 | | 3 | | 4 | | 5 | | Cannot assess | | |  |
| The current anti-doping rules are justified because it protects clean sport [NORMATIVE LEGITIMACY] | | | |  | | |  | |  | |  | |  | |  | | |  |
| The current anti-doping system is effective in protecting clean sport [PROCEDURAL LEGITIMACY: FAIR OUTCOME] | | | |  | | |  | |  | |  | |  | |  | | |  |
| The current anti-doping rules are implemented globally and equally (i.e. I believe that every athlete receives the same number of controls no matter where he/she comes from) [PROCEDURAL LEGITIMACY: FAIR PROCESS] | | | |  | | |  | |  | |  | |  | |  | | |  |
| Gender: | | □ female □ male | | | | | | | | | | | | | | | |  |
| Age: | | _____ years | | | | | | | | | | | | | | | |  |
| Country: | | _________________________________ | | | | | | | | | | | | | | | |  |
| Sport: | | _________________________________ | | | | | | | | | | | | | | | |  |
| Are you a member of a national testing pool? | | □ Yes □ No □ Don’t know | | | | | | | | | | | | | | | |  |
| Have you ever been selected for a doping control? | | □ Yes □ No | | | | | | | | | | | | | | | |  |
| If yes: | | □ In-competition □ Out-of-competition, Training □ Both | | | | | | | | | | | | | | | |  |

**Table** **S2**: *Adapted Assessment Tool –Phase Two*

| Have you ever received anti-doping education (e.g. lecture, info event etc.)? | | | | | | | | | | | | | | | | | | | | |
| --- | --- | --- | --- | --- | --- | --- | --- | --- | --- | --- | --- | --- | --- | --- | --- | --- | --- | --- | --- | --- |
| □ Yes  □ No | | | | | | | | | | | | | | | | | | | | |
| If yes, what were the contents of the education? (Multiple responses possible) | | | | | | | | | | | | | | | | | | | | |
| □ | Information about prohibited substances/methods | | | | | | | | | | | | | | | | | | | |
| □ | Information about anti-doping organizations and their responsibilities | | | | | | | | | | | | | | | | | | | |
| □ | Contents of the World Anti-Doping Code 2015 | | | | | | | | | | | | | | | | | | | |
| □ | Role plays (e.g. dilemma situations) | | | | | | | | | | | | | | | | | | | |
| □ | Online programs/apps | | | | | | | | | | | | | | | | | | | |
| □ | Discussions | | | | | | | | | | | | | | | | | | | |
| □ | Others: _________________________ | | | | | | | | | | | | | | | | | | | |
| Based on the doping-prevention education I received^[[2]](#footnote-2)^, I am confident that I can deal with the following situations without using prohibited substances and/or methods  (1 = not confident at all – 5 = very confident)^#^ [SELF-EFFICACY – INTERACTIVE LEGITIMACY] | | | | | 1 | | | | 2 | | | | 3 | | | | 4 | | | 5 |
| … explicit pressure from my environment (e.g. I was told by my coach, peer, sponsors, federation, club what to do) | | | | |  | | | |  | | | |  | | | |  | | |  |
| **… implicit pressure (e.g. I think/feel that this is what my coach, peers, sponsor, federation, club wants me to do)** | | | | |  | | | |  | | | |  | | | |  | | |  |
| … physical limitations (e.g. injuries, illness, fatigue, overtraining) | | | | |  | | | |  | | | |  | | | |  | | |  |
| … psychological and emotional limitations (e.g. perceived lack of progress, perceived lack of trust in my own performance) | | | | |  | | | |  | | | |  | | | |  | | |  |
| … life-/career-defining events (moving away from home, junior to big league) | | | | |  | | | |  | | | |  | | | |  | | |  |
| *… be successful in my sport while staying clean* | | | | |  | | | |  | | | |  | | | |  | | |  |
| Based on the doping-prevention education I received^[[3]](#footnote-3)^, I am confident that I …  (1 = not confident at all - 5 = very confident) ^#^ [SELF-EFFICACY –CRITICAL LEGITIMACY] | | | | | | 1 | | 2 | | | | 3 | | | | | 4 | | | 5 |
| **… know what to do in my role as an elite athlete** | | | | | |  | |  | | | |  | | | | |  | | |  |
| **… *can educate other athletes in anti-doping matters*** | | | | | |  | |  | | | |  | | | | |  | | |  |
| **… know how to take action (reporting doping, whistleblowing)** | | | | | |  | |  | | | |  | | | | |  | | |  |
| **… *would report a doped teammate*** | | | | | |  | |  | | | |  | | | | |  | | |  |
| On a scale from 1 – 5 (not at all – very well), how well do you know or feel informed about… [PERCEIVED KNOWLEDGE] | | | | 1 | | | | 2 | | | | 3 | | | | | 4 | | | 5 |
| … the prohibited list of the World Anti-Doping Agency of 2020 | | | |  | | | |  | | | |  | | | | |  | | |  |
| … therapeutic use exemptions | | | |  | | | |  | | | |  | | | | |  | | |  |
| ... your rights and responsibilities during a doping control | | | |  | | | |  | | | |  | | | | |  | | |  |
| ... ADAMS and the "Whereabout System" | | | |  | | | |  | | | |  | | | | |  | | |  |
| … the definition of doping | | | |  | | | |  | | | |  | | | | |  | | |  |
| … the health-related side-effects of doping substances and methods | | | |  | | | |  | | | |  | | | | |  | | |  |
| … your rights and responsibilities under the World Anti-Doping Code 2015 | | | |  | | | |  | | | |  | | | | |  | | |  |
| ... the consequences of doping based on the World Anti-Doping Code 2015 | | | |  | | | |  | | | |  | | | | |  | | |  |
| ... the consequences of doping beyond a ban from sport (e.g. criminal law, loss of sponsoring) | | | |  | | | |  | | | |  | | | | |  | | |  |
| According to the World Anti-Doping Code 2015… [KNOWLEDGE] | | | True | | | | | | | False | | | | | Do not know | | | | | |
| … you are not allowed to use or possess any prohibited substances and/or methods without a valid reason | | |  | | | | | | |  | | | | |  | | | | | |
| … you can be banned from sport if you violate the anti-doping regulations of the Code | | |  | | | | | | |  | | | | |  | | | | | |
| … prohibited substances and/or methods are allowed after consultation with a physician | | |  | | | | | | |  | | | | |  | | | | | |
| … you are allowed to take any medication if you are ill as long as they are prescribed by a physician | | |  | | | | | | |  | | | | |  | | | | | |
| … you are allowed to take any medication if you are ill | | |  | | | | | | |  | | | | |  | | | | | |
| … you have to actively participate during doping controls | | |  | | | | | | |  | | | | |  | | | | | |
| … you are not allowed to refuse a doping control | | |  | | | | | | |  | | | | |  | | | | | |
| … if you are part of a testing pool, you have to provide detailed information about your whereabouts (training, competition) | | |  | | | | | | |  | | | | |  | | | | | |
| … the term “doping” also includes possession, trading and trafficking of prohibited substances and/or methods | | |  | | | | | | |  | | | | |  | | | | | |
| … you alone are responsible for any substance within your body | | |  | | | | | | |  | | | | |  | | | | | |
| After being caught doping… | | | True | | | | | | | False | | | | | Do not know | | | | | |
| … you can be banned from your sport | | |  | | | | | | |  | | | | |  | | | | | |
| … you can be banned from participating in any sport | | |  | | | | | | |  | | | | |  | | | | | |
| … there might be additional consequences next to a ban from sports (i.e. legal consequences) | | |  | | | | | | |  | | | | |  | | | | | |
| I trust that the following organizations are capable of fulfilling their responsibilities  (1 = not at all – 5 = very much) [TRUST: ABILITY or CAPABILITY] | | | | 1 | | | 2 | | | | 3 | | | 4 | | 5 | | | Cannot assess | |
| **… your National Anti-Doping Organization (NADO)** | | | |  | | |  | | | |  | | |  | |  | | |  | |
| … the World Anti-Doping Agency (WADA) | | | |  | | |  | | | |  | | |  | |  | | |  | |
| … the International Olympic Committee (IOC) | | | |  | | |  | | | |  | | |  | |  | | |  | |
| … **my national sports federation** | | | |  | | |  | | | |  | | |  | |  | | |  | |
| … my international sports federation | | | |  | | |  | | | |  | | |  | |  | | |  | |
| I trust that the following organizations take care of people/athletes  (1 = not at all – 5 = very much) [TRUST: BENEVOLENCE] | | | | 1 | | | 2 | | | | 3 | | | 4 | | 5 | | | Cannot assess | |
| **… your National Anti-Doping Organization (NADO)** | | | |  | | |  | | | |  | | |  | |  | | |  | |
| … the World Anti-Doping Agency (WADA) | | | |  | | |  | | | |  | | |  | |  | | |  | |
| … the International Olympic Committee (IOC) | | | |  | | |  | | | |  | | |  | |  | | |  | |
| … **my national sports federation** | | | |  | | |  | | | |  | | |  | |  | | |  | |
| … my international sports federation | | | |  | | |  | | | |  | | |  | |  | | |  | |
| I trust that the following organizations keep their promises  (1 = not at all – 5 = very much) [TRUST:INTEGRITY] | | | | 1 | | | 2 | | | | 3 | | | 4 | | 5 | | | Cannot assess | |
| **… your National Anti-Doping Organization (NADO)** | | | |  | | |  | | | |  | | |  | |  | | |  | |
| … the World Anti-Doping Agency (WADA) | | | |  | | |  | | | |  | | |  | |  | | |  | |
| … the International Olympic Committee (IOC) | | | |  | | |  | | | |  | | |  | |  | | |  | |
| … **my national sports federation** | | | |  | | |  | | | |  | | |  | |  | | |  | |
| … my international sports federation | | | |  | | |  | | | |  | | |  | |  | | |  | |
| Please indicate the level of agreement with the following statements:  (1 = strongly disagree – 5 = strongly agree) [PERCEIVED LEGITIMACY] | | | | 1 | | | 2 | | | | 3 | | | 4 | | 5 | | Cannot assess | | |
| The current anti-doping rules are justified because it protects clean sport [NORMATIVE LEGITIMACY] | | | |  | | |  | | | |  | | |  | |  | |  | | |
| The current anti-doping system is effective in protecting clean sport [PROCEDURAL LEGITIMACY: FAIR OUTCOME] | | | |  | | |  | | | |  | | |  | |  | |  | | |
| The current anti-doping rules are implemented globally and equally (i.e. I believe that every athlete receives the same number of controls no matter where he/she comes from) [PROCEDURAL LEGITIMACY: FAIR PROCESS] | | | |  | | |  | | | |  | | |  | |  | |  | | |
| Gender: | | □ female □ male | | | | | | | | | | | | | | | | | | |
| Age: | | _____ years | | | | | | | | | | | | | | | | | | |
| Country: | | _________________________________ | | | | | | | | | | | | | | | | | | |
| Sport: | | _________________________________ | | | | | | | | | | | | | | | | | | |
| Are you a member of a national testing pool? | | □ Yes □ No □ Don’t know | | | | | | | | | | | | | | | | | | |
| Have you ever been selected for a doping control? | | □ Yes □ No | | | | | | | | | | | | | | | | | | |
| If yes: | | □ In-competition □ Training □ Both | | | | | | | | | | | | | | | | | | |

*Note: ^#^ Filter questions to either athletes who received or did not receive anti-doping education; bold: newly added content; bold and italics: changed content; italics and crossed out: deleted content*

**Table** **S 3**: *Descriptive Statistics of Overall Functional and Interactive Literacy and Separated by Perceived Level of Education (Phase One)*

|  | *Level of Edu* | *n* | *Mean* | *SD* | *95% CI* | | *Min* | *Max* | *F-Test/*  *Welch Test^#^* | *p* |
| --- | --- | --- | --- | --- | --- | --- | --- | --- | --- | --- |
| ***Functional Literacy – Perceived Knowledge*** | | | | | | | | | | |
| *Prohibited List* | *no edu* | *245* | *2.79* | *1.4* | *2.62* | *3.0* | *1* | *5* | *24.74* | *<.001^#^* |
|  | *info* | *290* | *3.52* | *1.1* | *3.39* | *3.7* | *1* | *5* |  |  |
|  | *ce* | *354* | *3.44** | *1.0* | *3.33* | *3.5* | *1* | *5* |  |  |
|  | ***Overall*** | ***889*** | ***3.29*** | ***1.2*** | ***3.21*** | ***3.4*** | ***1*** | ***5*** |  |  |
| *TUE* | *no edu* | *237* | *2.63* | *1.4* | *2.45* | *2.8* | *1* | *5* | *34.16* | *<.001^#^* |
|  | *info* | *281* | *3.53* | *1.2* | *3.39* | *3.7* | *1* | *5* |  |  |
|  | *ce* | *342* | *3.43** | *1.2* | *3.30* | *3.6* | *1* | *5* |  |  |
|  | ***Overall*** | ***860*** | ***3.24*** | ***1.3*** | ***3.15*** | ***3.3*** | ***1*** | ***5*** |  |  |
| *Rights and responsibilities during a doping control* | *no edu* | *243* | *3.18* | *1.4* | *3.00* | *3.4* | *1* | *5* | *59.74* | *<.001^#^* |
|  | *info* | *294* | *4.20* | *1.0* | *4.08* | *4.3* | *1* | *5* |  |  |
|  | *ce* | *356* | *4.31** | *0.9* | *4.21* | *4.4* | *1* | *5* |  |  |
|  | ***Overall*** | ***893*** | ***3.97*** | ***1.2*** | ***3.89*** | ***4.0*** | ***1*** | ***5*** |  |  |
| *ADAMS and Whereabout System* | *no edu* | *223* | *2.41* | *1.5* | *2.22* | *2.6* | *1* | *5* | *27.49* | *<.001^#^* |
|  | *info* | *274* | *3.28* | *1.4* | *3.12* | *3.5* | *1* | *5* |  |  |
|  | *ce* | *327* | *3.23** | *1.3* | *3.08* | *3.4* | *1* | *5* |  |  |
|  | ***Overall*** | ***824*** | ***3.03*** | ***1.4*** | ***2.93*** | ***3.1*** | ***1*** | ***5*** |  |  |
| *Definition of Doping* | *no edu* | *248* | *3.72* | *1.2* | *3.57* | *3.9* | *1* | *5* | *22.93* | *<.001^#^* |
|  | *info* | *289* | *4.24* | *1.0* | *4.13* | *4.3* | *1* | *5* |  |  |
|  | *ce* | *358* | *4.32** | *0.9* | *4.23* | *4.4* | *1* | *5* |  |  |
|  | ***Overall*** | ***895*** | ***4.13*** | ***1.1*** | ***4.06*** | ***4.2*** | ***1*** | ***5*** |  |  |
| *Health-related side effects* | *no edu* | *242* | *3.27* | *1.3* | *3.10* | *3.4* | *1* | *5* | *39.48* | *<.001^#^* |
|  | *info* | *287* | *4.02* | *1.0* | *3.91* | *4.1* | *1* | *5* |  |  |
|  | *ce* | *356* | *4.15** | *1.0* | *4.05* | *4.2* | *1* | *5* |  |  |
|  | ***Overall*** | ***885*** | ***3.87*** | ***1.2*** | ***3.79*** | ***3.9*** | ***1*** | ***5*** |  |  |
| *Rights and responsibilities WADC* | *no edu* | *231* | *2.79* | *1.5* | *2.60* | *3.0* | *1* | *5* | *35.46* | *<.001^#^* |
|  | *info* | *287* | *3.70* | *1.2* | *3.57* | *3.8* | *1* | *5* |  |  |
|  | *ce* | *342* | *3.68** | *1.2* | *3.56* | *3.8* | *1* | *5* |  |  |
|  | ***Overall*** | ***860*** | ***3.45*** | ***1.3*** | ***3.36*** | ***3.5*** | ***1*** | ***5*** |  |  |
| *Consequences of doping based on WADC* | *no edu* | *238* | *3.19* | *1.3* | *3.02* | *3.4* | *1* | *5* | *30.27* | *<.001^#^* |
|  | *info* | *283* | *3.86* | *1.2* | *3.71* | *4.0* | *1* | *5* |  |  |
|  | *ce* | *352* | *3.99** | *1.1* | *3.88* | *4.1* | *1* | *5* |  |  |
|  | ***Overall*** | ***873*** | ***3.73*** | ***1.2*** | ***3.65*** | ***3.8*** | ***1*** | ***5*** |  |  |
| *Consequences doping beyond sport* | *no edu* | *235* | *3.18* | *1.4* | *3.00* | *3.4* | *1* | *5* | *34.52* | *<.001^#^* |
|  | *info* | *285* | *3.88* | *1.2* | *3.74* | *4.0* | *1* | *5* |  |  |
|  | *ce* | *358* | *4.08** | *1.1* | *3.96* | *4.2* | *1* | *5* |  |  |
|  | ***Overall*** | ***878*** | ***3.77*** | ***1.3*** | ***3.69*** | ***3.9*** | ***1*** | ***5*** |  |  |
| ***Functional Literacy – Test Knowledge*** | | | | | | | | | | |
| *WADC* | *no edu* | *268* | *0.56* | *.29* | *.53* | *.60* | *0* | *1* | *16.45* | *<.001* |
|  | *info* | *299* | *0.63** | *.28* | *.60* | *.66* | *0* | *1* |  |  |
|  | *ce* | *363* | *.69*/*** | *.26* | *.66* | *.72* | *0* | *1* |  |  |
|  | ***Overall*** | ***930*** | ***.64*** | ***.28*** | ***.62*** | ***.65*** | ***0*** | ***1*** |  |  |
| *Consequences Doping* | *no edu* | *267* | *.65* | *.33* | *.61* | *.69* | *0* | *1* | *19.04* | *<.001^#^* |
|  | *info* | *296* | *.73** | *.33* | *.69* | *.77* | *0* | *1* |  |  |
|  | *ce* | *362* | *.80*/*** | *.26* | *.77* | *.83* | *0* | *1* |  |  |
|  | ***Overall*** | ***925*** | ***.74*** | ***.31*** | ***.72*** | ***.76*** | ***0*** | ***1*** |  |  |
| ***Interactive Literacy*** | | | | | | | | | | |
| *Confidence explicit pressure* | *no edu* | *266* | *4.17* | *1.3* | *4.00* | *4.3* | *1* | *5* | *5.81* | *0.003^#^* |
|  | *info* | *294* | *4.33* | *1.1* | *4.21* | *4.5* | *1* | *5* |  |  |
|  | *ce* | *346* | *4.49** | *1.0* | *4.38* | *4.6* | *1* | *5* |  |  |
|  | ***Overall*** | ***906*** | ***4.34*** | ***1.1*** | ***4.27*** | ***4.4*** | ***1*** | ***5*** |  |  |
| *Confidence physical limitations* | *no edu* | *262* | *4.00* | *1.3* | *3.85* | *4.2* | *1* | *5* | *8.65* | *<.001^#^* |
|  | *info* | *286* | *4.19* | *1.1* | *4.06* | *4.3* | *1* | *5* |  |  |
|  | *ce* | *349* | *4.38** | *0.9* | *4.28* | *4.5* | *1* | *5* |  |  |
|  | ***Overall*** | ***897*** | ***4.21*** | ***1.1*** | ***4.14*** | ***4.3*** | ***1*** | ***5*** |  |  |
| *Confidence psychological and emotional limitations* | *no edu* | *264* | *4.02* | *1.3* | *3.86* | *4.2* | *1* | *5* | *7.01* | *0.001^#^* |
|  | *info* | *294* | *4.19* | *1.1* | *4.06* | *4.3* | *1* | *5* |  |  |
|  | *ce* | *353* | *4.38** | *1.0* | *4.27* | *4.5* | *1* | *5* |  |  |
|  | ***Overall*** | ***911*** | ***4.21*** | ***1.2*** | ***4.14*** | ***4.3*** | ***1*** | ***5*** |  |  |
| *Confidence life-/career-defining events* | *no edu* | *263* | *4.21* | *1.3* | *4.05* | *4.4* | *1* | *5* | *3.97* | *0.019^#^* |
|  | *info* | *290* | *4.24* | *1.1* | *4.12* | *4.4* | *1* | *5* |  |  |
|  | *ce* | *347* | *4.44** | *1.0* | *4.33* | *4.5* | *1* | *5* |  |  |
|  | ***Overall*** | ***900*** | ***4.31*** | ***1.1*** | ***4.24*** | ***4.4*** | ***1*** | ***5*** |  |  |
| *Confidence being successful in my sport* | *no edu* | *269* | *4.46* | *1.1* | *4.33* | *4.6* | *1* | *5* | *8.44* | *<.001^#^* |
|  | *info* | *295* | *4.61* | *0.9* | *4.51* | *4.7* | *1* | *5* |  |  |
|  | *ce* | *351* | *4.75*/*** | *0.7* | *4.69* | *4.8* | *1* | *5* |  |  |
|  | ***Overall*** | ***915*** | ***4.62*** | ***0.9*** | ***4.56*** | ***4.7*** | ***1*** | ***5*** |  |  |

*Note: #: Welch Test was applied (in these cases Tahmane post-hoc tests were applied); * significant difference compared to no education; ** significant difference compared to information only; SD: standard deviation; n: number of respondents; CI: Confidence Interval, Min: Minimum; Max: Maximum; PK: perceived knowledge, ce: comprehensive education*

**Table *S4:*** *Descriptive Statistics of Overall Legitimacy and Trust and Separated by Perceived Level of Education (Phase One)*

|  |  | *n* | *Mean* | *SD* | *95% CI* | | *Min* | *Max* | *F-Test/*  *Welch Test^#^* | *p* |
| --- | --- | --- | --- | --- | --- | --- | --- | --- | --- | --- |
| *Perceived Legitimacy: The current anti-doping…* | | | | | | | | | | |
| .. rules are justified because it protects clean sport | no edu | 220 | 4.29 | 1.1 | 4.15 | 4.4 | 1 | 5 | 8.63 | <.001^#^ |
|  | info | 271 | 4.49 | 0.8 | 4.39 | 4.6 | 2 | 5 |  |  |
|  | ce | 338 | 4.62* | 0.7 | 4.54 | 4.7 | 1 | 5 |  |  |
|  | **Overall** | **829** | **4.49** | **0.9** | **4.43** | **4.5** | **1** | **5** |  |  |
| … system is effective in protecting clean sport | no edu | 219 | 4.02 | 1.1 | 3.88 | 4.2 | 1 | 5 | 11.140 | 0.003 |
|  | info | 269 | 4.28* | 0.9 | 4.17 | 4.4 | 1 | 5 |  |  |
|  | ce | 336 | 4.29* | 1.0 | 4.18 | 4.4 | 1 | 5 |  |  |
|  | **Overall** | **824** | **4.22** | **1.0** | **4.15** | **4.3** | **1** | **5** |  |  |
| … rules are implemented globally and equally | no edu | 211 | 3.71 | 1.3 | 3.53 | 3.9 | 1 | 5 | 2.68 | 0.069^#^ |
|  | info | 261 | 3.97 | 1.1 | 3.83 | 4.1 | 1 | 5 |  |  |
|  | ce | 326 | 3.92 | 1.3 | 3.78 | 4.1 | 1 | 5 |  |  |
|  | **Overall** | **798** | **3.88** | **1.2** | **3.79** | **4.0** | **1** | **5** |  |  |
| *Perceived Trust* | | | | | | | | | | |
| WADA: Capability | no edu | 227 | 4.15 | 1.1 | 4.01 | 4.29 | 1.0 | 5.0 | 8.75 | <.001^#^ |
|  | info | 276 | 4.30 | 1.0 | 4.18 | 4.41 | 1.0 | 5.0 |  |  |
|  | ce | 339 | 4.49*/** | 0.8 | 4.40 | 4.58 | 1.0 | 5.0 |  |  |
|  | **Overall** | **842** | **4.33** | **1.0** | **4.27** | **4.40** | **1.0** | **5.0** |  |  |
| WADA: Member Concern | no edu | 217 | 4.14 | 1.0 | 4.01 | 4.27 | 1.0 | 5.0 | 3.52 | .030 |
|  | info | 268 | 4.26 | 1.0 | 4.15 | 4.38 | 1.0 | 5.0 |  |  |
|  | ce | 333 | 4.36* | 0.9 | 4.26 | 4.45 | 1.0 | 5.0 |  |  |
|  | **Overall** | **818** | **4.27** | **0.9** | **4.20** | **4.33** | **1.0** | **5.0** |  |  |
| WADA: Keeping the promise | no edu | 218 | 4.01 | 1.1 | 3.87 | 4.16 | 1.0 | 5.0 | 10.99 | <.001 |
|  | info | 263 | 4.30* | 0.9 | 4.19 | 4.41 | 1.0 | 5.0 |  |  |
|  | ce | 333 | 4.39* | 0.9 | 4.30 | 4.49 | 1.0 | 5.0 |  |  |
|  | **Overall** | **814** | **4.26** | **1.0** | **4.19** | **4.33** | **1.0** | **5.0** |  |  |
| IOC: Capabilty | no edu | 229 | 4.28 | 1.0 | 4.14 | 4.41 | 1.0 | 5.0 | 3.53 | .030^#^ |
|  | info | 274 | 4.33 | 1.0 | 4.22 | 4.45 | 1.0 | 5.0 |  |  |
|  | ce | 334 | 4.47* | 0.9 | 4.38 | 4.57 | 1.0 | 5.0 |  |  |
|  | ***Overall*** | **837** | **4.37** | **0.9** | **4.31** | **4.44** | **1.0** | **5.0** |  |  |
| IOC: Member Concern | no edu | 224 | 4.22 | 1.0 | 4.09 | 4.35 | 1.0 | 5.0 | 2.24 | .11 |
|  | info | 266 | 4.27 | 1.0 | 4.16 | 4.39 | 1.0 | 5.0 |  |  |
|  | ce | 333 | 4.38 | 0.9 | 4.29 | 4.48 | 1.0 | 5.0 |  |  |
|  | ***Overall*** | **823** | **4.30** | **0.9** | **4.24** | **4.37** | **1.0** | **5.0** |  |  |
| IOC: Keeping the promise | no edu | 221 | 4.19 | 1.0 | 4.05 | 4.32 | 1.0 | 5.0 | 2.36 | .10 |
|  | info | 267 | 4.30 | 0.9 | 4.19 | 4.41 | 1.0 | 5.0 |  |  |
|  | ce | 335 | 4.36 | 0.9 | 4.26 | 4.46 | 1.0 | 5.0 |  |  |
|  | ***Overall*** | **823** | **4.29** | **0.9** | **4.23** | **4.36** | **1.0** | **5.0** |  |  |
| NF: Capabilty | no edu | 229 | 3.91 | 1.2 | 3.75 | 4.06 | 1.0 | 5.0 | 4.45 | .012^#^ |
|  | info | 275 | 4.13 | 1.1 | 4.01 | 4.26 | 1.0 | 5.0 |  |  |
|  | ce | 336 | 4.19* | 1.0 | 4.08 | 4.30 | 1.0 | 5.0 |  |  |
|  | ***Overall*** | **840** | **4.09** | **1.1** | **4.02** | **4.17** | **1.0** | **5.0** |  |  |
| NF: Member Concern | no edu | 226 | 3.98 | 1.1 | 3.83 | 4.13 | 1.0 | 5.0 | 4.41 | .018^#^ |
|  | info | 269 | 4.20 | 1.0 | 4.08 | 4.33 | 1.0 | 5.0 |  |  |
|  | ce | 333 | 4.23* | 1.0 | 4.12 | 4.34 | 1.0 | 5.0 |  |  |
|  | ***Overall*** | ***828*** | ***4.15*** | ***1.1*** | ***4.08*** | ***4.23*** | ***1.0*** | ***5.0*** |  |  |
| NF: Keeping the promise | no edu | 228 | 3.79 | 1.2 | 3.62 | 3.95 | 1.0 | 5.0 | 9.86 | <.001^#^ |
|  | info | 268 | 4.16* | 1.0 | 4.03 | 4.28 | 1.0 | 5.0 |  |  |
|  | ce | 334 | 4.21* | 1.0 | 4.11 | 4.32 | 1.0 | 5.0 |  |  |
|  | ***Overall*** | **830** | **4.08** | **1.1** | **4.00** | **4.15** | **1.0** | **5.0** |  |  |
| IF: Capabilty | no edu | 223 | 4.08 | 1.0 | 3.94 | 4.22 | 1.0 | 5.0 | 4.01 | .018 |
|  | info | 272 | 4.17 | 1.0 | 4.05 | 4.29 | 1.0 | 5.0 |  |  |
|  | ce | 332 | 4.32* | 0.9 | 4.21 | 4.42 | 1.0 | 5.0 |  |  |
|  | ***Overall*** | **827** | **4.21** | **1.0** | **4.14** | **4.27** | **1.0** | **5.0** |  |  |
| IF: Member Concern | no edu | 220 | 4.11 | 1.0 | 3.98 | 4.25 | 1.0 | 5.0 | 3.11 | .046^#^ |
|  | info | 266 | 4.22 | 1.0 | 4.10 | 4.34 | 1.0 | 5.0 |  |  |
|  | ce | 325 | 4.32* | 0.9 | 4.22 | 4.42 | 1.0 | 5.0# |  |  |
|  | ***Overall*** | **811** | **4.23** | **1.0** | **4.17** | **4.30** | **1.0** | **5.0** |  |  |
| IF: Keeping the promise | no edu | 219 | 4.01 | 1.1 | 3.86 | 4.16 | 1.0 | 5.0 | 5.04 | .007^#^ |
|  | info | 266 | 4.18 | 0.9 | 4.06 | 4.29 | 1.0 | 5.0 |  |  |
|  | ce | 325 | 4.29* | 0.9 | 4.19 | 4.39 | 1.0 | 5.0 |  |  |
|  | ***Overall*** | **810** | **4.18** | **1.0** | **4.11** | **4.25** | **1.0** | **5.0** |  |  |

*Note: #: Welch Test was applied (in these cases Tahmane post-hoc tests were applied); * significant difference compared to no education; ** significant difference compared to information only; SD: standard deviation; n: number of respondents; CI: Confidence Interval, Min: Minimum; Max: Maximum;* *ce: comprehensive education*

**Table** **S5**: *Differences in ADL between different levels of perceived anti-doping education (Phase Two)*

|  | | N | Mean | SD | 95% CI | | Min | Max | F-Test/  Welch Test^#^ | p |
| --- | --- | --- | --- | --- | --- | --- | --- | --- | --- | --- |
| **Functional Literacy – Perceived Knowledge** | | | | | | | | | | |
| Prohibited List | no edu | 298 | 2.73 | 1.2 | 2.59 | 2.9 | 1 | 5 | 43.14 | <.001 |
|  | info | 233 | 3.24* | 1.2 | 3.09 | 3.4 | 1 | 5 |  |  |
|  | ce | 661 | 3.49*/** | 1.1 | 3.40 | 3.6 | 1 | 5 |  |  |
|  | Overall | 1192 | 3.25 | 1.2 | 3.18 | 3.3 | 1 | 5 |  |  |
| TUE | no edu | 295 | 2.59 | 1.3 | 2.44 | 2.7 | 1 | 5 | 54.88 | <.001 |
|  | info | 231 | 3.20* | 1.2 | 3.04 | 3.4 | 1 | 5 |  |  |
|  | ce | 655 | 3.49*/** | 1.2 | 3.40 | 3.6 | 1 | 5 |  |  |
|  | Overall | 1181 | 3.21 | 1.3 | 3.14 | 3.3 | 1 | 5 |  |  |
| Rights and responsibilities during a doping control | no edu | 297 | 3.24 | 1.3 | 3.09 | 3.4 | 1 | 5 | 66.72 | <.001^#^ |
|  | info | 229 | 3.92* | 1.1 | 3.77 | 4.1 | 1 | 5 |  |  |
|  | ce | 660 | 4.23*/** | 1.0 | 4.16 | 4.3 | 1 | 5 |  |  |
|  | Overall | 1186 | 3.93 | 1.2 | 3.86 | 4.0 | 1 | 5 |  |  |
| ADAMS and "Whereabout System" | no edu | 295 | 2.53 | 1.4 | 2.37 | 2.7 | 1 | 5 | 28.33 | <.001 |
|  | info | 232 | 2.93* | 1.4 | 2.75 | 3.1 | 1 | 5 |  |  |
|  | ce | 657 | 3.25*/** | 1.4 | 3.15 | 3.4 | 1 | 5 |  |  |
|  | Overall | 1184 | 3.01 | 1.4 | 2.93 | 3.1 | 1 | 5 |  |  |
| Definition of Doping | no edu | 297 | 3.77 | 1.2 | 3.64 | 3.9 | 1 | 5 | 24.06 | <.001^#^ |
|  | info | 233 | 4.08* | 1.0 | 3.96 | 4.2 | 1 | 5 |  |  |
|  | ce | 655 | 4.30*/** | 0.9 | 4.23 | 4.4 | 1 | 5 |  |  |
|  | Overall | 1185 | 4.13 | 1.0 | 4.07 | 4.2 | 1 | 5 |  |  |
| Health-related side effects | no edu | 296 | 3.42 | 1.3 | 3.27 | 3.6 | 1 | 5 | 31.11 | <.001^#^ |
|  | info | 228 | 3.84* | 1.1 | 3.70 | 4.0 | 1 | 5 |  |  |
|  | ce | 660 | 4.10*/** | 1.0 | 4.02 | 4.2 | 1 | 5 |  |  |
|  | Overall | 1184 | 3.88 | 1.2 | 3.81 | 3.9 | 1 | 5 |  |  |
| Rights and responsibilities WADC | no edu | 295 | 2.73 | 1.3 | 2.58 | 2.9 | 1 | 5 | 52.74 | <.001^#^ |
|  | info | 231 | 3.38* | 1.2 | 3.22 | 3.5 | 1 | 5 |  |  |
|  | ce | 658 | 3.65*/** | 1.2 | 3.56 | 3.7 | 1 | 5 |  |  |
|  | Overall | 1184 | 3.37 | 1.3 | 3.30 | 3.4 | 1 | 5 |  |  |
| consequences of doping based on WADC | no edu | 294 | 2.94 | 1.3 | 2.79 | 3.1 | 1 | 5 | 53.31 | <.001^#^ |
|  | info | 230 | 3.71* | 1.2 | 3.55 | 3.9 | 1 | 5 |  |  |
|  | ce | 657 | 3.8* | 1.2 | 3.78 | 4.0 | 1 | 5 |  |  |
|  | Overall | 1181 | 3.61 | 1.3 | 3.53 | 3.7 | 1 | 5 |  |  |
| consequences doping beyond sport | no edu | 295 | 3.22 | 1.4 | 3.06 | 3.4 | 1 | 5 | 37.80 | <.001^#^ |
|  | info | 232 | 3.79* | 1.2 | 3.63 | 3.9 | 1 | 5 |  |  |
|  | ce | 656 | 4.03*/** | 1.1 | 3.94 | 4.1 | 1 | 5 |  |  |
|  | Overall | 1183 | 3.78 | 1.3 | 3.71 | 3.9 | 1 | 5 |  |  |
| **Functional Literacy – Test Knowledge** | | | | | | | | | | |
| Knowledge WADC | no edu | 297 | 5.42 | 2.6 | 5.12 | 5.7 | 0 | 10 | 29.19 | <.001 |
|  | info | 229 | 6.41* | 2.7 | 6.05 | 6.8 | 0 | 10 |  |  |
|  | ce | 655 | 6.83* | 2.6 | 6.63 | 7.0 | 0 | 10 |  |  |
|  | Overall | 1181 | 6.39 | 2.7 | 6.24 | 6.5 | 0 | 10 |  |  |
| Knowledge Consequences Doping | no edu | 286 | 1.77 | 0.8 | 1.67 | 1.9 | 0 | 3 | 11.61 | <.001^#^ |
|  | info | 225 | 1.93 | 0.8 | 1.83 | 2.0 | 0 | 3 |  |  |
|  | ce | 640 | 2.05* | 0.8 | 1.99 | 2.1 | 0 | 3 |  |  |
|  | Overall | 1151 | 1.95 | 0.8 | 1.91 | 2.0 | 0 | 3 |  |  |
| **Interactive Literacy** | | | | | | | | | | |
| Confidence explicit pressure | no edu | 304 | 4.05 | 1.3 | 3.91 | 4.2 | 1 | 5 | 9.36 | <.001^#^ |
|  | info | 240 | 4.09 | 1.2 | 3.93 | 4.2 | 1 | 5 |  |  |
|  | ce | 664 | 4.36*/** | 1.0 | 4.28 | 4.4 | 1 | 5 |  |  |
|  | Overall | 1208 | 4.23 | 1.2 | 4.16 | 4.3 | 1 | 5 |  |  |
| Confidence implicit pressure | no edu | 303 | 4.00 | 1.3 | 3.85 | 4.1 | 1 | 5 | 7.87 | <.001^#^ |
|  | info | 240 | 4.08 | 1.2 | 3.93 | 4.2 | 1 | 5 |  |  |
|  | ce | 664 | 4.30*/** | 1.1 | 4.22 | 4.4 | 1 | 5 |  |  |
|  | Overall | 1207 | 4.18 | 1.2 | 4.12 | 4.2 | 1 | 5 |  |  |
| Confidence physical limitations | no edu | 302 | 3.91 | 1.3 | 3.76 | 4.1 | 1 | 5 | 11.49 | <.001^#^ |
|  | info | 237 | 3.99 | 1.2 | 3.84 | 4.1 | 1 | 5 |  |  |
|  | ce | 662 | 4.27*/** | 1.1 | 4.19 | 4.4 | 1 | 5 |  |  |
|  | Overall | 1201 | 4.12 | 1.2 | 4.06 | 4.2 | 1 | 5 |  |  |
| Confidence psychological and emotional limitations | no edu | 292 | 3.85 | 1.3 | 3.69 | 4.0 | 1 | 5 | 14.69 | <.001^#^ |
|  | info | 239 | 3.94 | 1.3 | 3.78 | 4.1 | 1 | 5 |  |  |
|  | ce | 662 | 4.27*/** | 1.1 | 4.19 | 4.4 | 1 | 5 |  |  |
|  | Overall | 1193 | 4.10 | 1.2 | 4.03 | 4.2 | 1 | 5 |  |  |
| Confidence life-/career-defining events | no edu | 293 | 3.95 | 1.4 | 3.79 | 4.1 | 1 | 5 | 8.51 | <.001^#^ |
|  | info | 226 | 4.04 | 1.3 | 3.88 | 4.2 | 1 | 5 |  |  |
|  | ce | 643 | 4.30*/** | 1.1 | 4.21 | 4.4 | 1 | 5 |  |  |
|  | Overall | 1162 | 4.16 | 1.2 | 4.09 | 4.2 | 1 | 5 |  |  |
| Confidence what to do in my role as an elite athlete | no edu | 295 | 4.49 | 0.8 | 4.40 | 4.6 | 2 | 5 | 2.76 | .064^#^ |
|  | info | 231 | 4.49 | 0.7 | 4.39 | 4.6 | 2 | 5 |  |  |
|  | ce | 637 | 4.59 | 0.7 | 4.53 | 4.6 | 2 | 5 |  |  |
|  | Overall | 1163 | 4.54 | 0.7 | 4.50 | 4.6 | 2 | 5 |  |  |
| **Critical Literacy** | | | | | | | | | | |
| Confidence educate other athletes | no edu | 255 | 3.62 | 1.0 | 3.49 | 3.7 | 2 | 5 | 14.40 | <.001 |
|  | info | 207 | 3.61 | 1.1 | 3.46 | 3.8 | 2 | 5 |  |  |
|  | ce | 620 | 3.95*/** | 1.0 | 3.87 | 4.0 | 2 | 5 |  |  |
|  | Overall | 1082 | 3.81 | 1.0 | 3.74 | 3.9 | 2 | 5 |  |  |
| Confidence to take action (report doping, whistleblow) | no edu | 254 | 3.71 | 1.1 | 3.58 | 3.8 | 2 | 5 | 16.70 | <.001^#^ |
|  | info | 209 | 4.04* | 0.9 | 3.92 | 4.2 | 2 | 5 |  |  |
|  | ce | 626 | 4.17* | 1.0 | 4.09 | 4.2 | 2 | 5 |  |  |
|  | Overall | 1089 | 4.04 | 1.0 | 3.98 | 4.1 | 2 | 5 |  |  |
| Confidence to report a doped teammate | no edu | 238 | 3.76 | 1.1 | 3.62 | 3.9 | 2 | 5 | 9.56 | <.001^#^ |
|  | info | 193 | 3.99 | 1.0 | 3.84 | 4.1 | 2 | 5 |  |  |
|  | ce | 585 | 4.11* | 1.0 | 4.03 | 4.2 | 2 | 5 |  |  |
|  | Overall | 1016 | 4.01 | 1.0 | 3.94 | 4.1 | 2 | 5 |  |  |

*Note: #: Welch Test was applied (in these cases Tahmane post-hoc tests were applied); * significant difference compared to no education; ** significant difference compared to information only; SD: standard deviation; n: number of respondents; CI: Confidence Interval, Min: Minimum; Max: Maximum; PK: perceived knowledge; ce: comprehensive education*

**Table** **S6**: *Differences in Legitimacy and Trust between different levels of perceived anti-doping education (Phase Two)*

|  |  | n | Mean | SD | 95% CI | | Min | Max | F-Test/  Welch Test^#^ | p |
| --- | --- | --- | --- | --- | --- | --- | --- | --- | --- | --- |
| *Perceived Legitimacy: The current anti-doping…* | | | | | | | | | | |
| .. rules are justified because it protects clean sport | no edu | 226 | 4.37 | 1.1 | 4.23 | 4.5 | 1 | 5 | 3.51 | .031^#^ |
|  | info | 199 | 4.49 | 1.0 | 4.35 | 4.6 | 1 | 5 |  |  |
|  | ce | 559 | 4.57* | 0.8 | 4.50 | 4.6 | 1 | 5 |  |  |
|  | Overall | 984 | 4.51 | 0.9 | 4.45 | 4.6 | 1 | 5 |  |  |
| … system is effective in protecting clean sport | no edu | 214 | 4.06 | 1.2 | 3.90 | 4.2 | 1 | 5 | 3.06 | .048^#^ |
|  | info | 195 | 4.20 | 1.0 | 4.06 | 4.3 | 1 | 5 |  |  |
|  | ce | 555 | 4.28* | 1.0 | 4.20 | 4.4 | 1 | 5 |  |  |
|  | Overall | 964 | 4.22 | 1.0 | 4.15 | 4.3 | 1 | 5 |  |  |
| … rules are implemented globally and equally | no edu | 204 | 3.71 | 1.4 | 3.51 | 3.9 | 1 | 5 | 1.86 | .16 |
|  | info | 176 | 3.57 | 1.3 | 3.39 | 3.8 | 1 | 5 |  |  |
|  | ce | 518 | 3.79 | 1.3 | 3.68 | 3.9 | 1 | 5 |  |  |
|  | Overall | 898 | 3.73 | 1.3 | 3.64 | 3.8 | 1 | 5 |  |  |
| Perceived Trust | | | | | | | | | | |
| NADA: Capability | no edu | 210 | 4.05 | 1.0 | 3.91 | 4.19 | 1 | 5 | 9.50 | <.001^#^ |
|  | info | 193 | 4.27 | 0.9 | 4.14 | 4.40 | 1 | 5 |  |  |
|  | ce | 581 | 4.39* | 0.9 | 4.32 | 4.46 | 1 | 5 |  |  |
|  | Overall | 984 | 4.29 | 0.9 | 4.24 | 4.35 | 1 | 5 |  |  |
| NADA: Member Concern | no edu | 202 | 4.10 | 1.0 | 3.96 | 4.23 | 1 | 5 | 7.55 | .001^#^ |
|  | info | 190 | 4.21 | 1.0 | 4.07 | 4.35 | 1 | 5 |  |  |
|  | ce | 569 | 4.38* | 0.9 | 4.31 | 4.45 | 1 | 5 |  |  |
|  | Overall | 961 | 4.29 | 0.9 | 4.23 | 4.34 | 1 | 5 |  |  |
| NADA: Keeping the promise | no edu | 208 | 4.05 | 1.0 | 3.91 | 4.18 | 1 | 5 | 11.71 | <.001 |
|  | info | 182 | 4.27 | 1.0 | 4.13 | 4.41 | 1 | 5 |  |  |
|  | ce | 562 | 4.41* | 0.9 | 4.33 | 4.48 | 1 | 5 |  |  |
|  | Overall | 952 | 4.30 | 0.9 | 4.24 | 4.36 | 1 | 5 |  |  |
| WADA: Capability | no edu | 221 | 4.20 | 1.0 | 4.07 | 4.33 | 1 | 5 | 4.81 | .008 |
|  | info | 200 | 4.34 | 0.9 | 4.21 | 4.46 | 1 | 5 |  |  |
|  | ce | 589 | 4.42* | 0.9 | 4.35 | 4.49 | 1 | 5 |  |  |
|  | Overall | 1010 | 4.36 | 0.9 | 4.30 | 4.41 | 1 | 5 |  |  |
| WADA: Member Concern | no edu | 210 | 4.25 | 0.9 | 4.12 | 4.37 | 1 | 5 | 2.10 | .12 |
|  | info | 196 | 4.28 | 0.9 | 4.16 | 4.40 | 1 | 5 |  |  |
|  | ce | 588 | 4.38 | 0.9 | 4.31 | 4.45 | 1 | 5 |  |  |
|  | Overall | 994 | 4.33 | 0.9 | 4.28 | 4.39 | 1 | 5 |  |  |
| WADA: Keeping the promise | no edu | 216 | 4.15 | 0.9 | 4.02 | 4.27 | 1 | 5 | 8.02 | <.001 |
|  | info | 191 | 4.30 | 1.0 | 4.16 | 4.44 | 1 | 5 |  |  |
|  | ce | 581 | 4.43* | 0.9 | 4.36 | 4.50 | 1 | 5 |  |  |
|  | Overall | 988 | 4.34 | 0.9 | 4.29 | 4.40 | 1 | 5 |  |  |
| IOC: Capability | no edu | 222 | 4.29 | 1.0 | 4.16 | 4.42 | 1 | 5 | 1.89 | .15 |
|  | info | 196 | 4.30 | 0.9 | 4.16 | 4.43 | 1 | 5 |  |  |
|  | ce | 585 | 4.41 | 0.9 | 4.33 | 4.48 | 1 | 5 |  |  |
|  | Overall | 1003 | 4.36 | 0.9 | 4.30 | 4.42 | 1 | 5 |  |  |
| IOC: Member Concern | no edu | 214 | 4.31 | 0.9 | 4.19 | 4.43 | 1 | 5 | 2.85 | .06 |
|  | info | 200 | 4.25 | 0.9 | 4.13 | 4.37 | 1 | 5 |  |  |
|  | ce | 588 | 4.41 | 0.9 | 4.34 | 4.48 | 1 | 5 |  |  |
|  | Overall | 1002 | 4.36 | 0.9 | 4.30 | 4.41 | 1 | 5 |  |  |
| IOC: Keeping the promise | no edu | 223 | 4.16 | 1.0 | 4.04 | 4.29 | 1 | 5 | 9.42 | <.001^#^ |
|  | info | 192 | 4.23 | 1.0 | 4.09 | 4.37 | 1 | 5 |  |  |
|  | ce | 584 | 4.45*/** | 0.9 | 4.38 | 4.51 | 1 | 5 |  |  |
|  | Overall | 999 | 4.34 | 0.9 | 4.28 | 4.40 | 1 | 5 |  |  |
| NF: Capabilty | no edu | 227 | 3.98 | 1.1 | 3.84 | 4.13 | 1 | 5 | 6.74 | .001 |
|  | info | 201 | 4.13 | 1.0 | 3.99 | 4.28 | 1 | 5 |  |  |
|  | ce | 590 | 4.27* | 1.0 | 4.19 | 4.35 | 1 | 5 |  |  |
|  | Overall | 1018 | 4.18 | 1.0 | 4.12 | 4.24 | 1 | 5 |  |  |
| NF: Member Concern | no edu | 225 | 4.11 | 1.1 | 3.97 | 4.25 | 1 | 5 | 7.06 | .001^#^ |
|  | info | 202 | 4.19 | 1.0 | 4.05 | 4.33 | 1 | 5 |  |  |
|  | ce | 598 | 4.37* | 0.9 | 4.30 | 4.45 | 1 | 5 |  |  |
|  | Overall | 1025 | 4.28 | 1.0 | 4.22 | 4.34 | 1 | 5 |  |  |
| NF: Keeping the promise | no edu | 227 | 4.00 | 1.1 | 3.85 | 4.14 | 1 | 5 | 8.06 | <.001 |
|  | info | 198 | 4.08 | 1.1 | 3.93 | 4.23 | 1 | 5 |  |  |
|  | ce | 589 | 4.29*/** | 1.0 | 4.21 | 4.37 | 1 | 5 |  |  |
|  | Overall | 1014 | 4.19 | 1.0 | 4.12 | 4.25 | 1 | 5 |  |  |
| IF: Capabilty | no edu | 214 | 4.14 | 1.0 | 4.01 | 4.28 | 1 | 5 | 4.77 | .009^#^ |
|  | info | 188 | 4.28 | 0.9 | 4.15 | 4.41 | 1 | 5 |  |  |
|  | ce | 561 | 4.38* | 0.9 | 4.31 | 4.45 | 1 | 5 |  |  |
|  | Overall | 963 | 4.31 | 0.9 | 4.25 | 4.37 | 1 | 5 |  |  |
| IF: Member Concern | no edu | 221 | 4.14 | 1.0 | 4.00 | 4.27 | 1 | 5 | 6.58 | .002^#^ |
|  | info | 196 | 4.22 | 0.9 | 4.10 | 4.35 | 1 | 5 |  |  |
|  | ce | 583 | 4.39* | 0.9 | 4.32 | 4.46 | 1 | 5 |  |  |
|  | Overall | 1000 | 4.30 | 0.9 | 4.24 | 4.36 | 1 | 5 |  |  |
| IF: Keeping the promise | no edu | 219 | 4.12 | 1.0 | 3.99 | 4.26 | 1 | 5 | 10.10 | <.001^#^ |
|  | info | 192 | 4.20 | 1.0 | 4.06 | 4.34 | 1 | 5 |  |  |
|  | ce | 579 | 4.43*/** | 0.8 | 4.36 | 4.50 | 1 | 5 |  |  |
|  | Overall | 990 | 4.32 | 0.9 | 4.26 | 4.38 | 1 | 5 |  |  |

*Note: #: Welch Test was applied (in these cases Tahmane post-hoc tests were applied); * significant difference compared to no cation; ** significant difference compared to information only; SD: standard deviation; n: number of respondents; CI: Confidence Interval, Min: Minimum; Max: Maximum; PK: perceived knowledge; ce: comprehensive education*

**Table S7**: *Descriptive Statistics of Functional, Interactive and Critical DAL and Separated by Congruence of Education Provision and Perception (Phase Two)*

|  | *Level of Edu* | *n* | *Mean* | *SD* | *95% CI* | | *Min* | *Max* | *F-Test* | *p* |
| --- | --- | --- | --- | --- | --- | --- | --- | --- | --- | --- |
| ***Functional Literacy – Perceived Knowledge*** | | | | | | | | | | |
| *Prohibited List* | *Info- nothing* | 214 | 2.73 | 1.2 | 2.57 | 2.89 | 1 | 5 | *30.69* | *<.001* |
|  | *Info-Info* | 91 | 3.13* | 1.3 | 2.87 | 3.40 | 1 | 5 |  |  |
|  | *Ce- info/nothing* | 216 | 3.09* | 1.2 | 2.93 | 3.26 | 1 | 5 |  |  |
|  | *Ce-Ce* | 395 | 3.64*/**/*** | 1.1 | 3.53 | 3.74 | 1 | 5 |  |  |
| *TUE* | *Info- nothing* | 211 | 2.51 | 1.3 | 2.34 | 2.68 | 1 | 5 | *43.89* | *<.001* |
|  | *Info-Info* | 90 | 3.01* | 1.2 | 2.76 | 3.26 | 1 | 5 |  |  |
|  | *Ce- info/nothing* | 215 | 3.12* | 1.3 | 2.95 | 3.30 | 1 | 5 |  |  |
|  | *Ce-Ce* | 393 | 3.66*/**/*** | 1.1 | 3.55 | 3.77 | 1 | 5 |  |  |
| *Rights and responsibilities during a doping control* | *Info- nothing* | 213 | 3.26 | 1.3 | 3.08 | 3.44 | 1 | 5 | *45.96* | *<.001* |
|  | *Info-Info* | 89 | 3.69* | 1.3 | 3.41 | 3.96 | 1 | 5 |  |  |
|  | *Ce- info/nothing* | 214 | 3.74* | 1.2 | 3.58 | 3.91 | 1 | 5 |  |  |
|  | *Ce-Ce* | 394 | 4.34*/**/*** | 0.9 | 4.26 | 4.43 | 1 | 5 |  |  |
| *ADAMS and "Whereabout System"* | *Info- nothing* | 212 | 2.59 | 1.4 | 2.40 | 2.78 | 1 | 5 | *18.86* | *<.001* |
|  | *Info-Info* | 91 | 2.97 | 1.4 | 2.69 | 3.25 | 1 | 5 |  |  |
|  | *Ce- info/nothing* | 214 | 2.69 | 1.4 | 2.50 | 2.88 | 1 | 5 |  |  |
|  | *Ce-Ce* | 394 | 3.36*/*** | 1.4 | 3.23 | 3.50 | 1 | 5 |  |  |
| *Definition of Doping* | *Info- nothing* | 213 | 3.79 | 1.2 | 3.64 | 3.95 | 1 | 5 | *21.36* | *<.001* |
|  | *Info-Info* | 90 | 4.13* | 0.9 | 3.94 | 4.32 | 1 | 5 |  |  |
|  | *Ce- info/nothing* | 217 | 3.93 | 1.1 | 3.78 | 4.07 | 1 | 5 |  |  |
|  | *Ce-Ce* | 392 | 4.41*/*** | 0.8 | 4.32 | 4.49 | 1 | 5 |  |  |
| *Health-related side effects* | *Info- nothing* | 212 | 3.51 | 1.3 | 3.34 | 3.69 | 1 | 5 | *26.69* | *<.001* |
|  | *Info-Info* | 88 | 3.77 | 1.1 | 3.54 | 4.01 | 1 | 5 |  |  |
|  | *Ce- info/nothing* | 214 | 3.63 | 1.2 | 3.46 | 3.79 | 1 | 5 |  |  |
|  | *Ce-Ce* | 394 | 4.25*/**/*** | 0.9 | 4.16 | 4.34 | 1 | 5 |  |  |
| *Rights and responsibilities WADC* | *Info- nothing* | 211 | 2.73 | 1.3 | 2.56 | 2.91 | 1 | 5 | *40.85* | *<.001* |
|  | *Info-Info* | 89 | 3.31* | 1.2 | 3.06 | 3.57 | 1 | 5 |  |  |
|  | *Ce- info/nothing* | 216 | 3.14* | 1.4 | 2.96 | 3.32 | 1 | 5 |  |  |
|  | *Ce-Ce* | 394 | 3.82*/**/*** | 1.1 | 3.72 | 3.93 | 1 | 5 |  |  |
| *Consequences of doping based on WADC* | *Info- nothing* | 210 | 2.91 | 1.3 | 2.73 | 3.09 | 1 | 5 | *46.53* | *<.001* |
|  | *Info-Info* | 89 | 3.51* | 1.3 | 3.24 | 3.77 | 1 | 5 |  |  |
|  | *Ce- info/nothing* | 215 | 3.52* | 1.3 | 3.34 | 3.69 | 1 | 5 |  |  |
|  | *Ce-Ce* | 394 | 4.08*/**/*** | 1.0 | 3.98 | 4.18 | 1 | 5 |  |  |
| *Consequences doping beyond sport* | *Info- nothing* | 211 | 3.23 | 1.4 | 3.04 | 3.41 | 1 | 5 | *32.76* | *<.001* |
|  | *Info-Info* | 91 | 3.65* | 1.3 | 3.39 | 3.91 | 1 | 5 |  |  |
|  | *Ce- info/nothing* | 215 | 3.62* | 1.3 | 3.45 | 3.80 | 1 | 5 |  |  |
|  | *Ce-Ce* | 393 | 4.20*/**/*** | 1.0 | 4.10 | 4.30 | 1 | 5 |  |  |
| ***Functional Literacy – Test Knowledge*** | | | | | | | | | | |
| *Knowledge WADC* | *Info- nothing* | 214 | 5.57 | 2.6 | 5.23 | 5.92 | 0 | 10 | *24.66* | *<.001* |
|  | *Info-Info* | 89 | 5.82 | 2.7 | 5.26 | 6.38 | 0 | 10 |  |  |
|  | *Ce- info/nothing* | 213 | 6.24* | 2.8 | 5.87 | 6.61 | 0 | 10 |  |  |
|  | *Ce-Ce* | 394 | 7.29*/**/*** | 2.4 | 7.05 | 7.52 | 0 | 10 |  |  |
| *Knowledge Consequences Doping* | *Info- nothing* | 206 | 1.76 | 0.8 | 1.65 | 1.87 | 0 | 3 | *10.41* | *<.001* |
|  | *Info-Info* | 86 | 1.84 | 0.9 | 1.65 | 2.02 | 0 | 3 |  |  |
|  | *Ce- info/nothing* | 209 | 1.93 | 0.8 | 1.82 | 2.04 | 0 | 3 |  |  |
|  | *Ce-Ce* | 384 | 2.11*/**/*** | 0.7 | 2.04 | 2.18 | 0 | 3 |  |  |
|  | | | | | | | | | | |
| *Confidence explicit pressure* | *Info- nothing* | 218 | 4.05 | 1.3 | 3.88 | 4.22 | 1 | 5 | *6.84* | *<.001* |
|  | *Info-Info* | 95 | 4.12 | 1.3 | 3.86 | 4.37 | 1 | 5 |  |  |
|  | *Ce- info/nothing* | 221 | 4.10 | 1.2 | 3.93 | 4.26 | 1 | 5 |  |  |
|  | *Ce-Ce* | 397 | 4.42*/*** | 1.0 | 4.33 | 4.52 | 1 | 5 |  |  |
| *Confidence implicit pressure* | *Info- nothing* | 217 | 3.99 | 1.3 | 3.81 | 4.16 | 1 | 217 | *9.14* | *<.001* |
|  | *Info-Info* | 94 | 4.00 | 1.3 | 3.73 | 4.27 | 1 | 94 |  |  |
|  | *Ce- info/nothing* | 222 | 4.14 | 1.1 | 3.98 | 4.29 | 1 | 222 |  |  |
|  | *Ce-Ce* | 398 | 4.43*/**/*** | 1.0 | 4.34 | 4.52 | 1 | 398 |  |  |
| Confidence physical limitations | *Info- nothing* | 216 | 3.90 | 1.3 | 3.72 | 4.08 | 1 | 5 | *10.23* | *<.001* |
|  | *Info-Info* | 95 | 3.98 | 1.2 | 3.73 | 4.23 | 1 | 5 |  |  |
|  | *Ce- info/nothing* | 218 | 4.00 | 1.2 | 3.84 | 4.17 | 1 | 5 |  |  |
|  | *Ce-Ce* | 397 | 4.37*/**/** | 1.0 | 4.28 | 4.47 | 1 | 5 |  |  |
| Confidence psychological and emotional limitations | *Info- nothing* | 208 | 3.82 | 1.4 | 3.63 | 4.01 | 1 | 5 | *11.40* | *<.001* |
|  | *Info-Info* | 94 | 4.06 | 1.3 | 3.81 | 4.32 | 1 | 5 |  |  |
|  | *Ce- info/nothing* | 219 | 3.92 | 1.3 | 3.75 | 4.08 | 1 | 5 |  |  |
|  | *Ce-Ce* | 396 | 4.35*/*** | 1.0 | 4.25 | 4.45 | 1 | 5 |  |  |
| Confidence life-/career-defining events | *Info- nothing* | 208 | 3.93 | 1.5 | 3.73 | 4.14 | 1 | 5 | *6.83* | *<.001* |
|  | *Info-Info* | 87 | 4.06 | 1.3 | 3.78 | 4.33 | 1 | 5 |  |  |
|  | *Ce- info/nothing* | 214 | 4.06 | 1.2 | 3.89 | 4.23 | 1 | 5 |  |  |
|  | *Ce-Ce* | 388 | 4.37*/*** | 1.0 | 4.27 | 4.47 | 1 | 5 |  |  |
| Confidence what to do in my role as an elite athlete | *Info- nothing* | 210 | 4.53 | 0.7 | 4.43 | 4.63 | 2 | 5 | *2.23* | *.08* |
|  | *Info-Info* | 89 | 4.60 | 0.7 | 4.45 | 4.74 | 2 | 5 |  |  |
|  | *Ce- info/nothing* | 217 | 4.42 | 0.8 | 4.32 | 4.52 | 2 | 5 |  |  |
|  | *Ce-Ce* | 385 | 4.57 | 0.7 | 4.50 | 4.65 | 2 | 5 |  |  |

*Note: * significant difference compared to info - nothing; ** significant difference compared to info-info;*** significant difference compared to ce-info/nothing; SD: standard deviation; n: number of respondents; CI: Confidence Interval. Min: Minimum; Max: Maximum; PK: perceived knowledge; ce: comprehensive education*

**Table** **S8**: *Descriptive Statistics of Trust and Legitimacy and Separated by Congruence of Education Provision and Perception (Phase Two)*

|  |  | *n* | *Mean* | *SD* | *95% CI* | | *Min* | *Max* | *F-Test/*  *Welch Test^#^* | *p* |
| --- | --- | --- | --- | --- | --- | --- | --- | --- | --- | --- |
| *Perceived Legitimacy: The current anti-doping…* | | | | | | | | | | |
| .. rules are justified because it protects clean sport | *Info- nothing* | 154 | 4.30 | 1.1 | 4.12 | 4.48 | 1 | 5 | 5.88 | .001 |
|  | *Info-Info* | 73 | 4.34 | 1.2 | 4.07 | 4.62 | 1 | 5 |  |  |
|  | *Ce- info/nothing* | 189 | 4.57 | 0.8 | 4.45 | 4.68 | 1 | 5 |  |  |
|  | *Ce-Ce* | 337 | 4.62 | 0.7 | 4.54 | 4.70 | 1 | 5 |  |  |
| … system is effective in protecting clean sport | *Info- nothing* | 151 | 3.94 | 1.3 | 3.73 | 4.15 | 1 | 5 | 6.40 | <.001 |
|  | *Info-Info* | 72 | 4.00 | 1.2 | 3.71 | 4.29 | 1 | 5 |  |  |
|  | *Ce- info/nothing* | 179 | 4.32 | 0.9 | 4.19 | 4.45 | 1 | 5 |  |  |
|  | *Ce-Ce* | 336 | 4.32 | 0.9 | 4.22 | 4.42 | 1 | 5 |  |  |
| … rules are implemented globally and equally | *Info- nothing* | 145 | 3.54 | 1.5 | 3.29 | 3.78 | 1 | 5 | 1.91 | .13 |
|  | *Info-Info* | 67 | 3.46 | 1.3 | 3.15 | 3.78 | 1 | 5 |  |  |
|  | *Ce- info/nothing* | 162 | 3.79 | 1.2 | 3.60 | 3.98 | 1 | 5 |  |  |
|  | *Ce-Ce* | 311 | 3.77 | 1.3 | 3.62 | 3.92 | 1 | 5 |  |  |
| *Perceived Trust* | | | | | | | | | | |
| NADA: Capability | *Info- nothing* | 148 | 4.05 | 1.0 | 3.90 | 4.21 | 1 | 5 | 10.45 | <.001 |
|  | *Info-Info* | 68 | 4.07 | 1.1 | 3.80 | 4.34 | 1 | 5 |  |  |
|  | *Ce- info/nothing* | 179 | 4.27 | 1.0 | 4.13 | 4.41 | 1 | 5 |  |  |
|  | *Ce-Ce* | 350 | 4.50*/**/*** | 0.8 | 4.41 | 4.59 | 1 | 5 |  |  |
| NADA: Member Concern | *Info- nothing* | 136 | 4.03 | 1.0 | 3.87 | 4.19 | 1 | 5 | 11.18 | <.001 |
|  | *Info-Info* | 68 | 4.07 | 1.0 | 3.83 | 4.32 | 1 | 5 |  |  |
|  | *Ce- info/nothing* | 181 | 4.27 | 0.9 | 4.13 | 4.40 | 1 | 5 |  |  |
|  | *Ce-Ce* | 344 | 4.49*/**/*** | 0.8 | 4.41 | 4.58 | 1 | 5 |  |  |
| NADA: Keeping the promise | *Info- nothing* | 142 | 3.99 | 1.0 | 3.81 | 4.16 | 1 | 5 | 13.36 | <.001 |
|  | *Info-Info* | 61 | 4.05 | 1.1 | 3.77 | 4.33 | 1 | 5 |  |  |
|  | *Ce- info/nothing* | 179 | 4.32* | 0.9 | 4.19 | 4.45 | 1 | 5 |  |  |
|  | *Ce-Ce* | 344 | 4.51*/** | 0.8 | 4.42 | 4.59 | 1 | 5 |  |  |
| WADA: Capability | *Info- nothing* | 158 | 4.16 | 1.0 | 4.01 | 4.31 | 1 | 5 | 3.53 | .015 |
|  | *Info-Info* | 73 | 4.18 | 1.0 | 3.94 | 4.42 | 1 | 5 |  |  |
|  | *Ce- info/nothing* | 182 | 4.38 | 0.8 | 4.26 | 4.50 | 1 | 5 |  |  |
|  | *Ce-Ce* | 352 | 4.42* | 1.0 | 4.32 | 4.52 | 1 | 5 |  |  |
| WADA: Member Concern | *Info- nothing* | 142 | 4.23 | 0.9 | 4.08 | 4.38 | 1 | 5 | 2.25 | .08 |
|  | *Info-Info* | 70 | 4.23 | 0.9 | 4.01 | 4.44 | 2 | 5 |  |  |
|  | *Ce- info/nothing* | 187 | 4.30 | 0.9 | 4.17 | 4.43 | 1 | 5 |  |  |
|  | *Ce-Ce* | 355 | 4.42 | 0.9 | 4.33 | 4.52 | 1 | 5 |  |  |
| WADA: Keeping the promise | *Info- nothing* | 148 | 4.12 | 1.0 | 3.96 | 4.28 | 1 | 5 | 5.92 | .001 |
|  | *Info-Info* | 65 | 4.12 | 1.1 | 3.85 | 4.40 | 1 | 5 |  |  |
|  | *Ce- info/nothing* | 186 | 4.32 | 0.9 | 4.20 | 4.45 | 1 | 5 |  |  |
|  | *Ce-Ce* | 351 | 4.46*/** | 0.9 | 4.36 | 4.55 | 1 | 5 |  |  |
| IOC: Capabilty | *Info- nothing* | 162 | 4.28 | 1.0 | 4.13 | 4.43 | 1 | 5 | 2.12 | .10 |
|  | *Info-Info* | 73 | 4.21 | 1.0 | 3.98 | 4.43 | 1 | 5 |  |  |
|  | *Ce- info/nothing* | 175 | 4.35 | 0.9 | 4.21 | 4.49 | 1 | 5 |  |  |
|  | *Ce-Ce* | 352 | 4.45 | 0.9 | 4.35 | 4.54 | 1 | 5 |  |  |
| IOC: Member Concern | *Info- nothing* | 149 | 4.25 | 1.0 | 4.09 | 4.40 | 1 | 5 | 3.02 | .03 |
|  | *Info-Info* | 74 | 4.20 | 0.9 | 4.00 | 4.41 | 2 | 5 |  |  |
|  | *Ce- info/nothing* | 184 | 4.34 | 0.9 | 4.21 | 4.46 | 1 | 5 |  |  |
|  | *Ce-Ce* | 356 | 4.46 | 0.9 | 4.37 | 4.54 | 1 | 5 |  |  |
| IOC: Keeping the promise | *Info- nothing* | 152 | 4.18 | 1.0 | 4.03 | 4.34 | 1 | 5 | 6.95 | <.001 |
|  | *Info-Info* | 69 | 4.03 | 1.1 | 3.77 | 4.29 | 1 | 5 |  |  |
|  | *Ce- info/nothing* | 187 | 4.26 | 0.9 | 4.13 | 4.39 | 1 | 5 |  |  |
|  | *Ce-Ce* | 353 | 4.47*/** | 0.9 | 4.38 | 4.56 | 1 | 5 |  |  |
| NF: Capabilty | *Info- nothing* | 160 | 3.95 | 1.1 | 3.78 | 4.12 | 1 | 5 | 6.67 | <.001 |
|  | *Info-Info* | 73 | 4.11 | 1.0 | 3.87 | 4.35 | 1 | 5 |  |  |
|  | *Ce- info/nothing* | 187 | 4.12 | 1.1 | 3.96 | 4.27 | 1 | 5 |  |  |
|  | *Ce-Ce* | 352 | 4.38*/*** | 0.9 | 4.29 | 4.48 | 1 | 5 |  |  |
| NF: Member Concern | *Info- nothing* | 154 | 4.03 | 1.1 | 3.86 | 4.21 | 1 | 5 | 10.95 | <.001 |
|  | *Info-Info* | 74 | 4.19 | 1.0 | 3.95 | 4.42 | 1 | 5 |  |  |
|  | *Ce- info/nothing* | 192 | 4.21 | 1.0 | 4.07 | 4.36 | 1 | 5 |  |  |
|  | *Ce-Ce* | 360 | 4.51*/** | 0.8 | 4.43 | 4.59 | 1 | 5 |  |  |
| NF: Keeping the promise | *Info- nothing* | 158 | 3.94 | 1.2 | 3.76 | 4.12 | 1 | 5 | 10.48 | <.001 |
|  | *Info-Info* | 71 | 4.03 | 1.1 | 3.76 | 4.29 | 1 | 5 |  |  |
|  | *Ce- info/nothing* | 188 | 4.11 | 1.1 | 3.95 | 4.26 | 1 | 5 |  |  |
|  | *Ce-Ce* | 357 | 4.42*/**/*** | 0.9 | 4.33 | 4.52 | 1 | 5 |  |  |
| IF: Capabilty | *Info- nothing* | 152 | 4.08 | 1.0 | 3.91 | 4.25 | 1 | 5 | 5.98 | <.001 |
|  | *Info-Info* | 68 | 4.21 | 1.0 | 3.97 | 4.44 | 1 | 5 |  |  |
|  | *Ce- info/nothing* | 174 | 4.32 | 0.8 | 4.19 | 4.44 | 1 | 5 |  |  |
|  | *Ce-Ce* | 342 | 4.44* | 0.9 | 4.35 | 4.53 | 1 | 5 |  |  |
| IF: Member Concern | *Info- nothing* | 152 | 4.05 | 1.1 | 3.88 | 4.22 | 1 | 5 | 10.26 | <.001 |
|  | *Info-Info* | 71 | 4.14 | 0.9 | 3.92 | 4.36 | 1 | 5 |  |  |
|  | *Edu- info/nothing* | 187 | 4.29 | 0.9 | 4.17 | 4.42 | 1 | 5 |  |  |
|  | *Edu-Edu* | 351 | 4.50*/** | 0.8 | 4.41 | 4.58 | 1 | 5 |  |  |
| IF: Keeping the promise | *Info- nothing* | 149 | 4.07 | 1.1 | 3.89 | 4.24 | 1 | 5 | 10.99 | <.001 |
|  | *Info-Info* | 68 | 4.06 | 1.1 | 3.80 | 4.32 | 1 | 5 |  |  |
|  | *Edu- info/nothing* | 186 | 4.26 | 0.9 | 4.12 | 4.39 | 1 | 5 |  |  |
|  | *Edu-Edu* | 348 | 4.51*/**/*** | 0.8 | 4.43 | 4.59 | 1 | 5 |  |  |

*Note: * significant difference compared to info - nothing; ** significant difference compared to info-info;*** significant difference compared to ce-info/nothing; SD: standard deviation; n: number of respondents; CI: Confidence Interval. Min: Minimum; Max: Maximum; ce: comprehensive education*

*
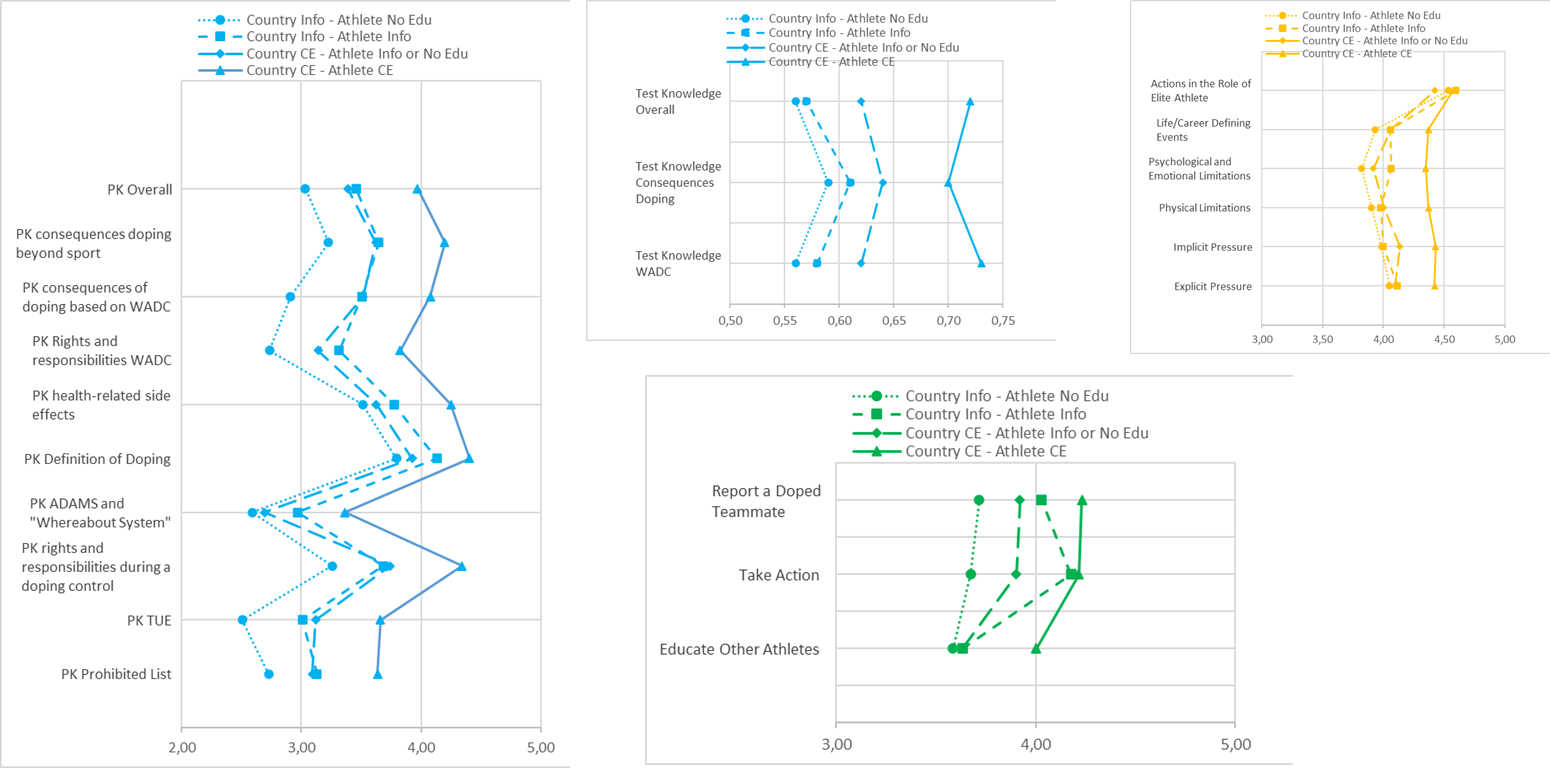
*

**Figure S1***: Differences in DAL between Congruence level of education reception and provision*

***Functional Literacy****: blue; Minimum – Maximum Perceived Knowledge:1 – 5; Minimum – Maximum Test Knowledge: 0 – 1 🡪 figures are scaled for more detailed information; PK: perceived knowledge, WADC: World Anti-Doping Code, TUE: Therapeutic Use Exemption, CE: comprehensive education; All changes significant for details on p-values and post hoc tests refer to table S7 in the supplemental material*

***Interactive Literacy:*** *orange; Minimum – Maximum:1 – 5; 🡪 figures are scaled for more detailed information; CE: comprehensive education; all changes significant for details on p-values and post hoc tests refer to table S7 in the supplemental material*

***Critical Literacy****: green; Minimum – Maximum:1 – 5; 🡪 figures are scaled for more detailed information; CE: comprehensive education; all changes significant for details on p-values and post hoc tests refer to table S7 in the supplemental material*


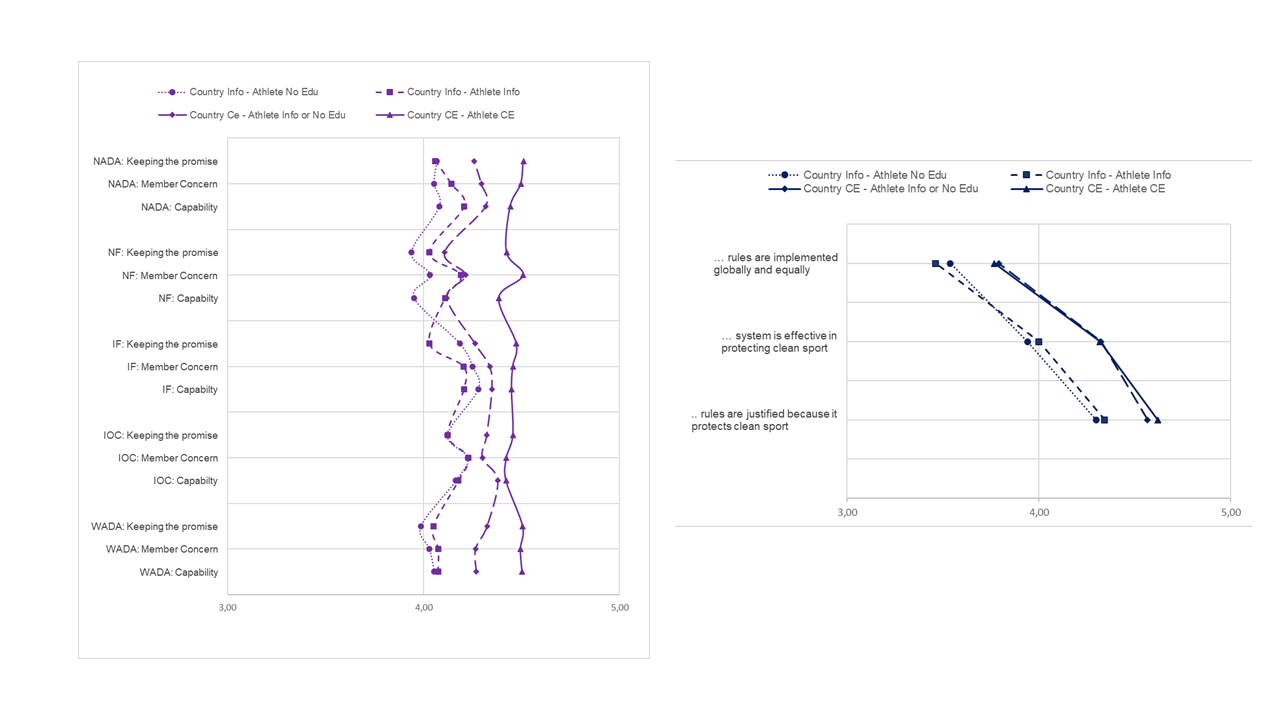


**Figure S2***: Differences in Perceived Trust and Legitimacy between Congruence level of education reception and provision*

***Trust:*** *purple; Minimum – Maximum:1 – 5; 🡪 figures are scaled for more detailed information; CE: comprehensive education; all changes significant for details on p-values and post hoc tests refer to table S8 in the supplemental material*

***Legitimacy:*** *dark blue; Minimum – Maximum 1 – 5; 🡪 figures are scaled for more detailed information; CE: comprehensive education, all changes significant for details on p-values and post hoc tests refer to table S8 in the supplemental material*

1. If respondents indicated they did not receive any form of anti-doping edudcation, the stem of that item changed to: *I am confident that I can deal with the following situations without using prohibited substances and/or methods* [↑](#footnote-ref-1)
2. If respondents indicated they did not receive any form of anti-doping edudcation, the stem of that item changed to: *I am confident that I can deal with the following situations without using prohibited substances and/or methods* [↑](#footnote-ref-2)
3. If respondents indicated they did not receive any form of anti-doping edudcation, the stem of that item changed to: *I am confident that I can deal with the following situations without using prohibited substances and/or methods* [↑](#footnote-ref-3)
